# A global decline in atmospheric dust during the 21st century

**DOI:** 10.1126/sciadv.aef5691

**Published:** 2026-09-16

**Authors:** Ashok Kumar Gupta, Jasper F. Kok, Vassilis Amiridis, Antonis Gkikas, Konstantinos Rizos, Emmanouil Proestakis, Stavros-Andreas Logothetis, Eleni Marinou, Carlos Pérez García-Pando

**Affiliations:** ^1^ Department of Earth and Environmental Sciences, Vanderbilt University, Nashville, TN, USA.; ^2^ Department of Atmospheric and Oceanic Sciences, University of California, Los Angeles, CA 90095, USA.; ^3^ Institute for Astronomy, Astrophysics, Space Applications and Remote Sensing, National Observatory of Athens, 15236 Athens, Greece.; ^4^ Research Centre for Atmospheric Physics and Climatology, Academy of Athens, 11521 Athens, Greece.; ^5^ Laboratory of Atmospheric Physics, Department of Physics, University of Patras, 26500 Patras, Greece.; ^6^ Barcelona Supercomputing Center, Barcelona, Spain.; ^7^ Catalan Institution for Research and Advanced Studies, Barcelona, Spain.

## Abstract

Mineral dust affects Earth’s energy budget, clouds, and biochemistry, yet the evolution of the global dust cycle in recent decades remains shrouded in uncertainty. Here we reconstruct the global dust cycle during the early 21st century by integrating a suite of global dust aerosol optical depth (DAOD) products from satellite-borne lidar, infrared sounders, and multispectral imagers; three aerosol reanalyses; and a compilation of dust-dominated AERONET sites into a large-ensemble inverse modeling framework. We show that North Africa has remained the dominant global dust source, yet it has experienced a decline in DAOD of −10 ± 8% between 2003 and 2023. A comparable reduction is observed across Asia (−10 ± 12%), together driving a global decrease of −10 ± 8%. Similar declines in dust loading, emissions, and deposition indicate a coherent reduction of the global dust cycle. Individual satellite products converge on overall similar trends, providing the strongest evidence to date that the planet has become less dusty in the early 21st century. This decline in atmospheric dust has exerted a positive effective radiative forcing of +0.02 ± 0.05 watts per square meter over 2003–2023, implying that declining atmospheric dust might have modestly contributed to the rapid warming of the past two decades.

## INTRODUCTION

Mineral dust is the most abundant natural aerosol by mass and influences Earth’s radiation budget, clouds, air quality, and biogeochemical cycles (*1*–*5*). However, how the global dust cycle has changed in the early 21st century remains poorly constrained, limiting assessments of dust’s contribution to recent warming. This is problematic because reductions in aerosols might have contributed to recent record-high global temperatures and the increase in Earth’s energy imbalance (*6*–*9*). Climate models also misrepresent the historical increase in atmospheric dust and its radiative impact (*3*, *10*), raising concerns about the reliability of simulated dust trends. Quantifying recent changes in dust aerosols, during the period when high-quality satellite and surface observations are available, is therefore necessary to constrain dust’s role in contemporary climate change (*7*).

Historical deposition records suggest that global dust mass loading has likely increased since preindustrial times (*3*). However, the sign and magnitude of dust trends over the past few decades remain uncertain (*10*). This uncertainty is amplified because many studies rely on individual satellite datasets or reanalyses with different sensitivities, spatial coverage, and evolving retrieval algorithms (*11*–*24*), making it difficult to distinguish a robust global increase or decrease in dust burden. Regional studies nonetheless point to consistent multidecadal declines in dust activity across East Asia (*25*) and North Africa (*26*), underscoring the need for a globally integrated assessment.

This uncertainty has direct implications for assessments of the historical aerosol effective radiative forcing (ERF) (*27*–*28*). Recent studies suggest that anthropogenic aerosol ERF has become less negative since around 2000, contributing to an acceleration of global warming and a strengthening of Earth’s energy imbalance (*6*, *7*, *29*, *30*). At the same time, Earth system models and many detection-attribution studies are typically poorly constrained with respect to the temporal evolution of aerosol forcing (*27*–*28*). If dust has changed substantially over the past two decades, its contribution to the net aerosol forcing, and thus to recent warming, may be misrepresented (*31*).

Here, we address this critical gap by developing a global, observationally constrained reconstruction of the dust cycle for the satellite era (2003–2023). By combining several state-of-the-art satellite-based dust aerosol optical depth (DAOD) products with targeted ground observations and a multimodel ensemble of global dust cycle simulations in an inverse modeling framework, we obtain global, internally consistent estimates of dust emission, atmospheric loading, DAOD, and deposition, with explicit propagation of uncertainties from DAOD retrievals, mass-extinction efficiency, and dust size distribution (Materials and Methods). This reconstruction yields regional and global trends and ready-to-use emission, loading, deposition, and DAOD datasets for climate and Earth-system modeling.

Over 2003–2023, we find that atmospheric dust declined in most regions, with column-integrated DAOD and dust loading decreasing globally by −5 ± 4% per decade, accompanied by comparable decreases in emission and deposition. This decrease in dust has enhanced decreases in aerosol optical depth (AOD) driven by declines in anthropogenic aerosol emission (*7*). As such, it might have contributed to both 21st century warming and the increase in Earth’s energy imbalance (*6*, *8*).

## RESULTS AND DISCUSSION

### Global DAOD trends from satellite and reanalysis products

**Fig. 1.**
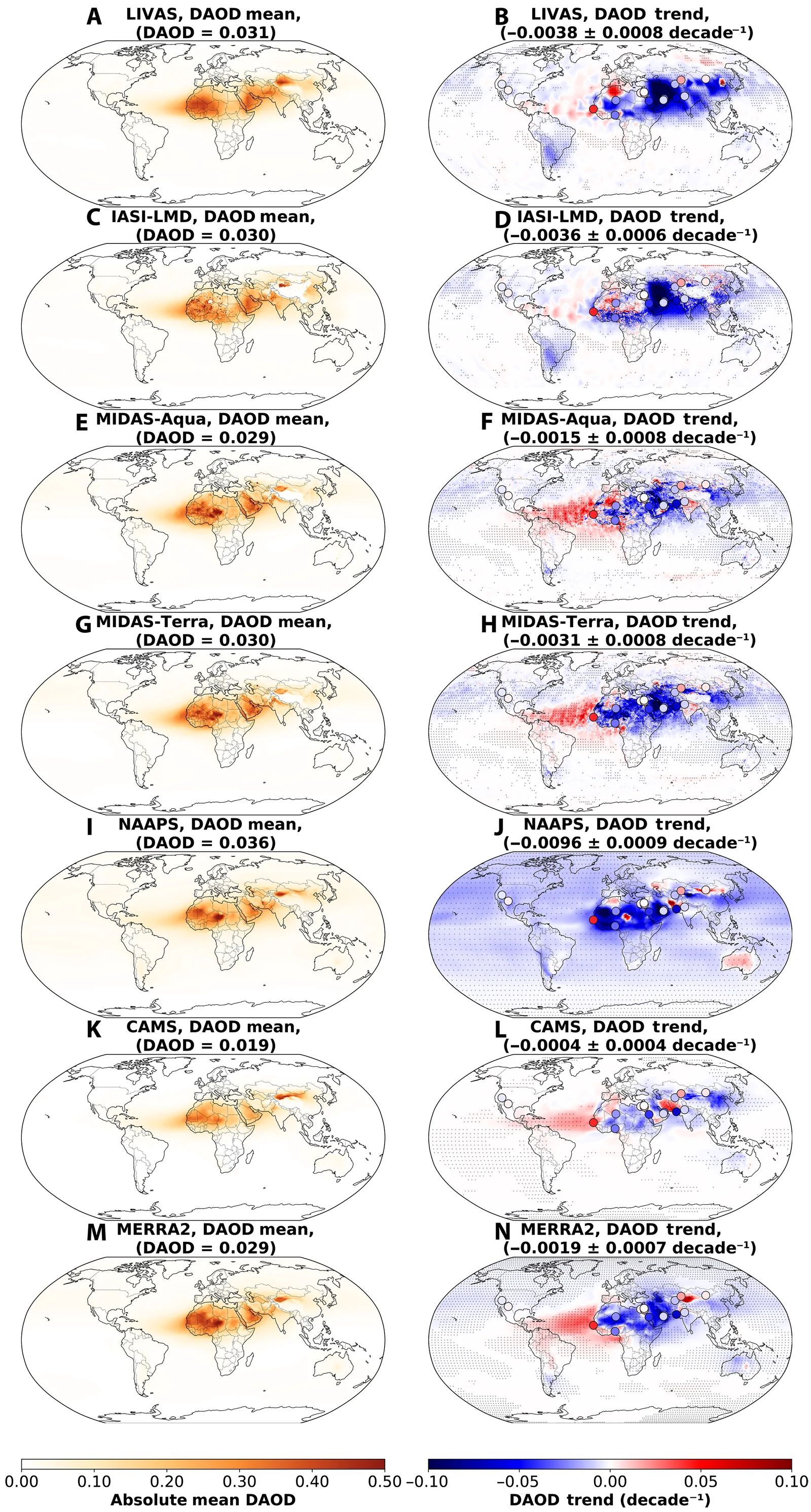
Global DAOD climatology and trends from satellite, reanalysis, and AERONET data. (**A,** **C**, **E**, **G**, **I**, **K**, and **M**) Annual mean DAOD at 550 nm (532 nm for LIVAS) for the overlapping period 2008–2021 from LIVAS (CALIPSO), IASI-LMD (LIVAS-fused), MIDAS-Aqua, MIDAS-Terra, NAAPS, CAMS-RA, and MERRA-2. (**B**, **D**, **F**, **H**, **J**, **L**, and **N**) Corresponding DAOD linear trends (decade^−1^). Circles in [(B), (D), (F), (H), (J), (L), and (N)] show the linear trends of conditionally filtered (see Materials and Methods) coarse-mode AOD derived from AERONET sun-photometer records at individual stations, as listed in table S2. Global area-weighted means and decadal trends are reported above each map. Satellite products converge to a global mean DAOD of ∼0.030 and exhibit consistent declines globally and over the major source regions; reanalysis products also show declines but with greater spread. Small gray triangles indicate grid cells where the linear DAOD trend is statistically significant at the 95% confidence level (*P* < 0.05).

We first quantity DAOD from four satellite-based observations [Lidar Climatology of Vertical Aerosol (LIVAS) (*13*), ModIs Dust AeroSol (MIDAS)–Aqua (*16*), MIDAS-Terra (*16*), and Infrared Atmospheric Sounding Interferometer–Laboratoire de Météorologie Dynamique (IASI-LMD) (*19*–*20*)] and three aerosol reanalysis products [MERRA-2 (*21*), Copernicus Atmosphere Monitoring Service (CAMS) (*22*), and Navy Aerosol Analysis and Prediction System (NAAPS) (*23*)] over their common period, 2008–2021 (Fig. 1, figs. S1 to S3, and table S1; Materials and Methods). The seven products yield a relatively narrow range of area-weighted global-mean DAOD (0.019 to 0.036), with the satellite ensemble converging at 0.030 ± 0.001, consistent with previous observational estimates of global DAOD (*16*, *32*–*33*). Among reanalyses, MERRA-2 aligns most closely with the satellite ensemble, whereas NAAPS is biased high and CAMS low, in agreement with recent multi-reanalysis intercomparisons (*5*). These products together offer a consistent assessment of long-term dust changes.

All seven products indicate a decline in global dust burden over 2008–2021. Global-mean DAOD trends are negative in every dataset, but the magnitude varies widely, from ∼−2% per decade in CAMS to nearly −30% per decade in NAAPS, with satellite products spanning ∼−5 to −25% per decade (fig. S4, last column). Despite this spread, the consistent sign across different observing and modeling systems supports a robust weakening of global dust aerosol loading.

DAOD has declined across the major North African and Middle Eastern source regions, whereas MODIS-based satellite products (MIDAS-Aqua and MIDAS-Terra) and reanalysis products (CAMS and MERRA-2) indicate a modest dust increase in the tropical North Atlantic dust outflow corridor (Fig. 1). These contrasting trends should be interpreted cautiously because the outflow corridor spans a sharp land-ocean DAOD gradient, and differences in the applied retrieval algorithms due to differences in the underlying surface type, cloud contamination, sampling, and aerosol partitioning can alias into regional trends (*13*, *16*, *18*). However, this source-outflow contrast could also point to a role for seasonally varying transport and removal in shaping the regional pattern (figs. S5 to S12) (*34*–*36*).

In DJF, the signal is more consistent across products, with a coherent positive trend over West Africa extending into the adjacent Atlantic (figs. S5 and S9). Intertropical convergence zone–linked precipitation, the principal wet-scavenging sink over the tropical Atlantic, offers a plausible physical pathway (*34*–*37*): Shifts in tropical rainfall can modulate wet-deposition efficiency and create an increase in dust over the outflow region even when source emissions decline (*38*–*39*). In JJA, LIVAS DAOD trends over the tropical North Atlantic exhibit a meridional gradient (fig. S7), with reductions across 15° to 25°N and increases between 5° and 15°N. The feature is most distinct in the lidar product and is not unambiguously reproduced by the passive-column retrievals or the reanalyses (fig. S7, J to N). The similar trends for IASI-LMD and LIVAS over the tropical North Atlantic occur because the IASI-LMD field outside the retained source-region mask is filled with LIVAS data, as described in Materials and Methods.

Climate model simulations indicate that under global warming, a shift toward less frequent but more intense precipitation reduces wet-deposition efficiency and can lead to increased atmospheric dust loading over outflow regions, even when source emissions decline (*40*, *41*). While this mechanism is physically plausible and supported by future-climate simulations (*41*), clear observational evidence for a detectable decrease in wet-scavenging efficiency in the tropical North Atlantic over the recent historical record remains limited.

Last, because a large fraction of the global dust burden is concentrated near major source regions, changes over North Africa exert a strong influence on the global DAOD tendency (Fig. 1 and fig. S4, last column). However, the sign of the North African trend is seasonally dependent (figs. S9 to S12). In DJF, most products indicate a positive DAOD trend over North African (aggregate) source regions, despite the negative annual mean trend (fig. S9). This wintertime enhancement indicates that the annual decline reflects seasonally heterogeneous changes in source-region emission and transport, rather than a uniform reduction.

### Evaluation of DAOD trends with AERONET

**Fig. 2.**
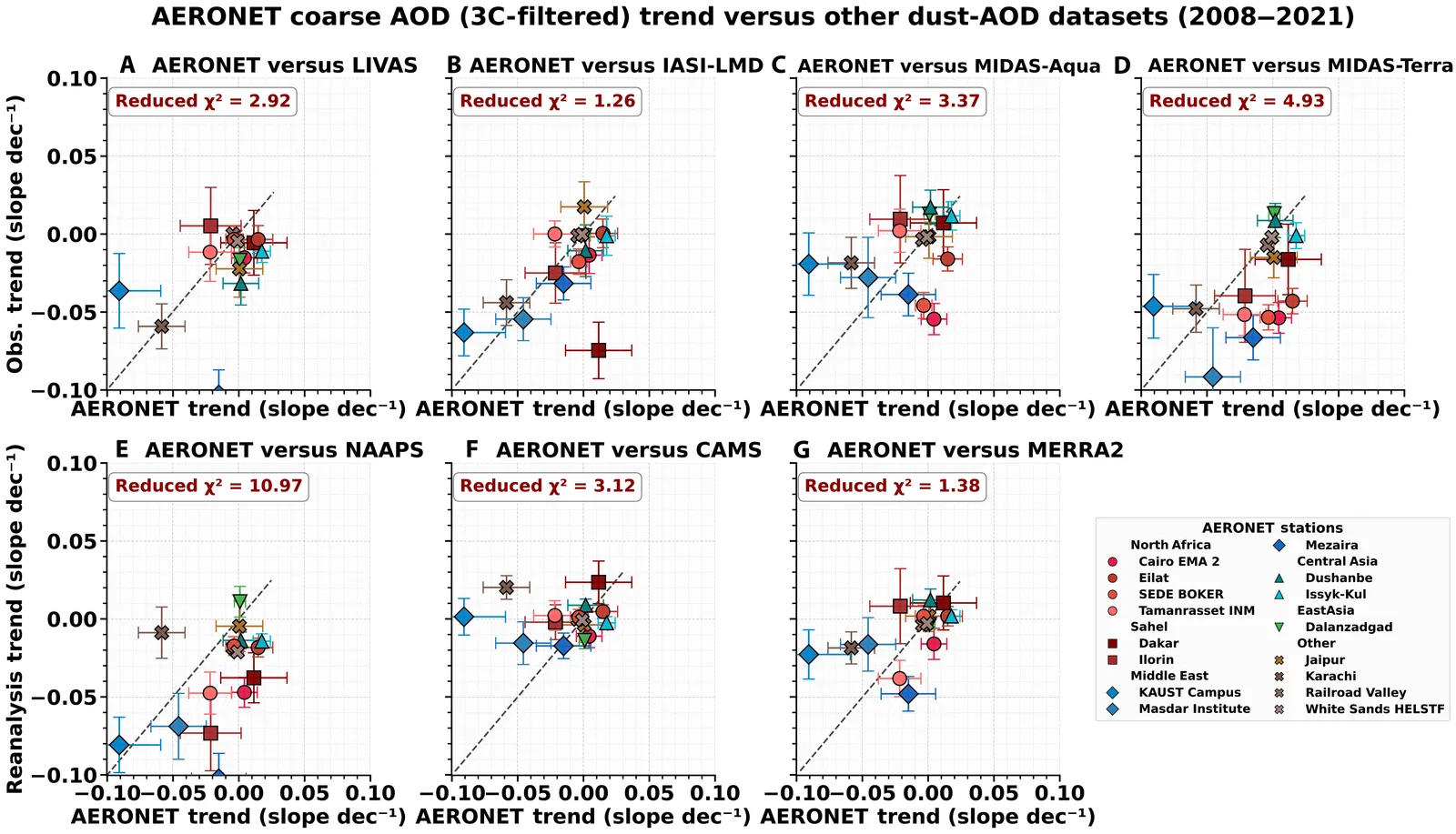
Evaluation of long-term DAOD trends of satellite and reanalysis products using AERONET data. Comparison of decadal DAOD trends (2008–2021) from each product with coarse-mode AOD trends derived at dust-dominated AERONET sites (Materials and Methods). Each panel shows the AERONET trend versus the product trend at the station location; error bars represent 1σ uncertainties. Reduced chi-square (χ^2^) values quantify consistency with AERONET (χ^2^ ≈ 1 indicates agreement within combined uncertainties). Overall agreement is mixed: IASI-LMD (LIVAS-fused) is close to χ^2^ ≈ 1; LIVAS and MIDAS-Terra tend to overestimate negative trends, whereas MIDAS-Aqua on balance underestimates them. Among reanalysis products, MERRA-2 shows good agreement, CAMS shows moderate agreement, and NAAPS exhibits the largest discrepancy.

We evaluate how well each DAOD product reproduces long-term dust trends by comparing 2008–2021 trends at dust-dominated AERONET stations using coarse-mode AOD (Fig. 2), which is commonly used as a proxy for DAOD (*42*). While several products show strong instantaneous agreement with AERONET (*43*), their skill in capturing decadal trends is less certain. Therefore, consistency is quantified with the reduced chi-square statistic (χ^2^), where χ^2^ ≈ 1 indicates agreement of decadal trends within combined uncertainties.

Among the seven products, IASI-LMD (χ^2^ = 1.26) and MERRA-2 (χ^2^ = 1.38) show the best trend consistency with AERONET. LIVAS and MIDAS-Terra generally overestimate negative trends, whereas MIDAS-Aqua tends to underestimate them, yielding larger χ^2^ values. CAMS shows moderate agreement, while NAAPS exhibits the largest biases, with trends typically far more negative than observed. Overall, IASI-LMD and MERRA-2 emerge as the most robust constraints on decadal dust variability. Their stronger performance aligns with the characteristics of their underlying observations. IASI uses thermal infrared radiances, where mineral dust has a clear spectral signature and bright desert surfaces are less problematic than in visible retrievals (*19*, *20*), supporting more accurate trend estimates; it is also preferentially sensitive to coarse dust, aligning well with AERONET coarse-mode AOD over source regions.

MERRA-2 benefits from assimilating multiple AOD records, which helps damp sensor-specific drifts and sampling discontinuities (*21*). By contrast, MODIS-based products are more exposed to platform-related effects (e.g., Terra overpass-time drift and calibration changes) (*24*) and to variability in fine-mode anthropogenic aerosol, both of which can alias into inferred dust trends.

### Observation-constrained reconstruction of the regional and global dust cycle

We therefore translate the DAOD changes observed over 2003–2023 in these various products into a reconstruction of the variability of the global dust cycle, including uncertainties, using an inverse modeling framework (*44*, *45*). This inverse model was originally developed to produce a 2004–2008 climatology of global dust emissions, loading, and DAOD by combining regional DAOD constraints with ensembles of chemical transport and climate model simulations (Materials and Methods).

Because satellite DAOD products differ in their long-term trends, we adopt an ensemble-of-observations approach wherever dust dominates the aerosol column, using all four calibrated satellite products (Materials and Methods). Regional and temporal bias corrections are applied to each satellite DAOD record using regional DAOD “ground truth” constraints from Ridley *et al.* (*32*). Ridley *et al.* (*32*) calibrated satellite AOD to AERONET AOD, used model simulations to separate dust from nondust AOD over dust-dominated regions, and quantified the uncertainty in the resulting regional DAOD. We propagate these uncertainties via bootstrap resampling, so that the spread across the inverse-model ensemble provides a robust measure of observational uncertainty (Materials and Methods).

**Fig. 3.**
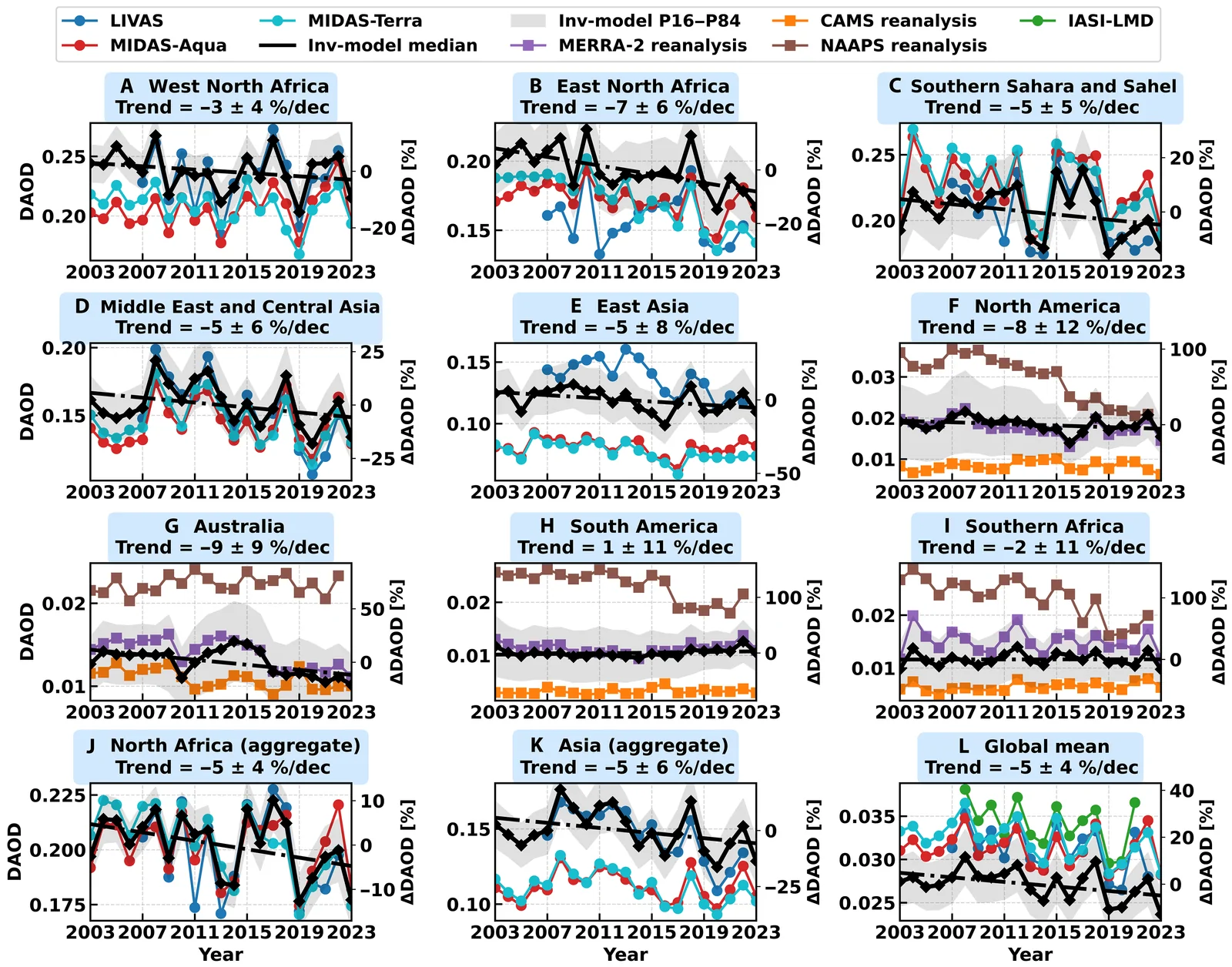
DAOD over the world’s main dust source regions from 2003 to 2023. Annual mean area-weighted DAOD for nine major dust source regions (see also fig. S2) (**A** to **I**), as well as for the aggregated North African (**J**) and Asian (**K**) source regions, and for the globe (**L**). Shown are the inverse model median (black line), its linear trend (dash-dotted line), and its one standard error (16th to 84th percentile) uncertainty range (shading). Satellite DAOD products are overlaid (colored lines) for the regions where AOD is dust dominated (in North African and Asian source regions), whereas three aerosol reanalysis products are shown in the four regions [(F) to (I)] where AOD is not dominated by dust. Both the satellite products and the inverse model show coherent decreases across the major dust source regions of North Africa and Asia, translating to a 10 ± 8% decrease in global DAOD from 2003 to 2023. For comparison, the corresponding annual mean satellite-derived DAOD trends over 2008–2021 are shown in fig. S4.

**Fig. 4.**
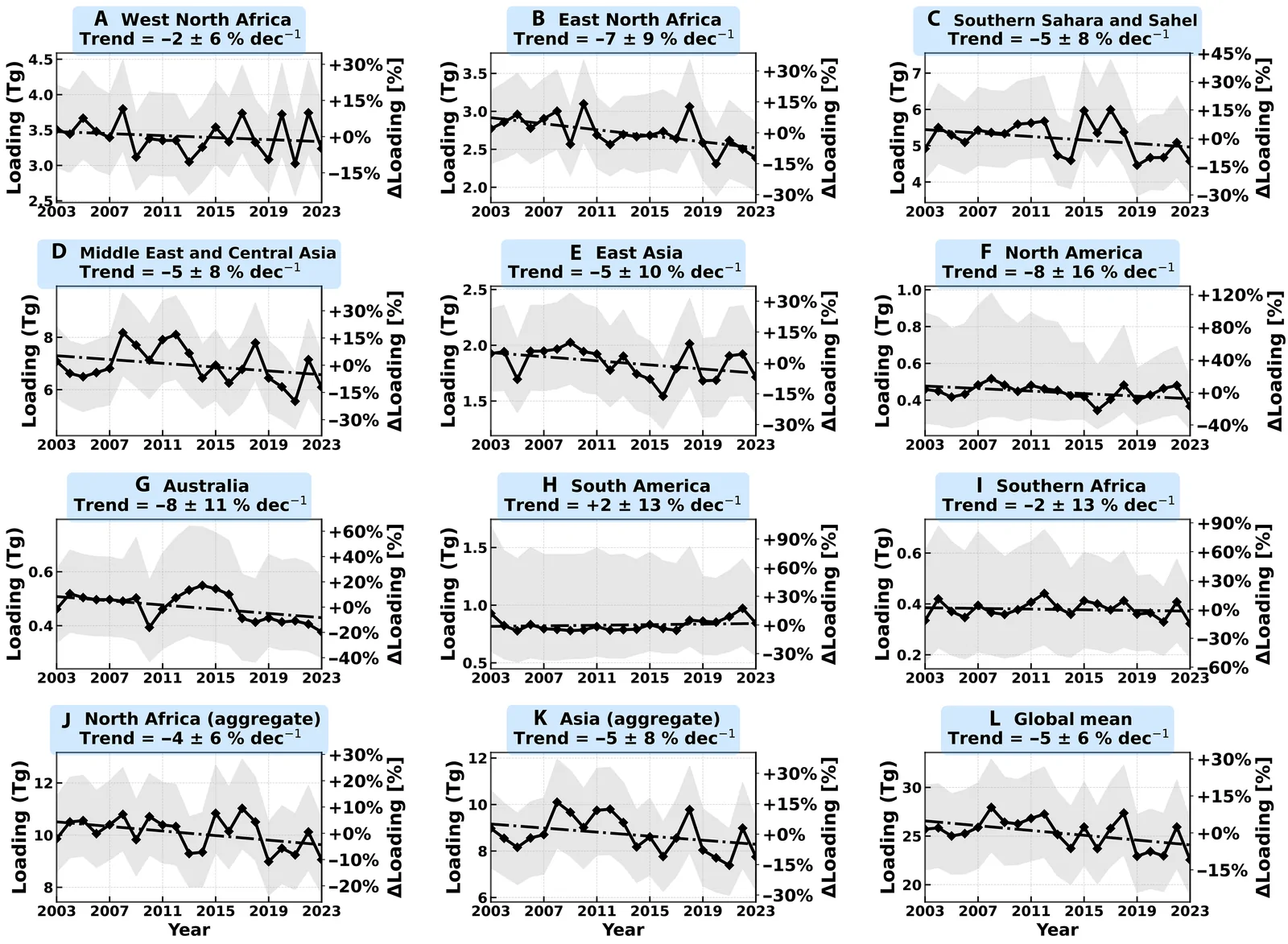
Inverse model–based reconstruction of dust loading from 2003 to 2023. Annual mean area-weighted dust loading over the nine dust-source regions (**A** to **I**) (fig. S1), over two aggregated domains over North Africa and Asia (**J** and **K**), and over the whole globe (**L**). The black line represents the inverse-model median, the dash-dotted line its linear fit, and the shading its one standard error range. The left *y* axis represents absolute loading, while the right *y* axis shows percentage changes relative to the 2003–2023 mean value. Similar to our results for DAOD (Fig. 3), global dust loading has decreased by 10 ± 12% from 2003 to 2023 due to decreases across the major dust source regions of North Africa and Asia.

We reconstruct the dust cycle for four consecutive ∼5-year periods, 2003–2008, 2009–2013, 2014–2018, and 2019–2023, starting with the first full year of joint MIDAS Terra-Aqua observations (*16*). Because DustCOMM requires ∼5-year averaging to reduce the influence of interannual meteorological variability (*46*), we use MERRA-2 dust fields to recover year-to-year variability within each period. This yields observation-constrained time series of DAOD, dust loading, and emission and deposition fluxes for nine major dust-source regions and for aggregated North African, Asian, and global domains (Figs. 3 and 4 and figs. S13 and S14).

The reconstructed DAOD time series show coherent decreases across North African and Asian source regions (Fig. 3, A to E), consistent with satellite and reanalysis products (Fig. 1) and AERONET station trends (Fig. 2). In regions where dust dominates column AOD, satellite-based decadal DAOD trends agree closely with the inverse-model ensemble median; in secondary regions where dust is a minor contributor, satellite-based DAOD is less reliable (*47*), and we rely on reanalysis products to constrain the inverse model dust cycle reconstruction (Fig. 3, F to I).

### Regional and global dust cycle changes

In North Africa (West North Africa, East North Africa, and the Southern Sahara and Sahel), multiproduct DAOD indicates domain-mean values of ∼0.16 to 0.22, and the inverse model yields a similar median range of 0.18 to 0.22 with 5 to 20% relative uncertainty (Fig. 3, A to C). The reconstructed DAOD time series shows a gradual decline with superimposed variability, reaching a minimum around 2019, rebounding in 2022, and returning to low values in 2023, consistent with recent evidence for reduced dust activity in parts of the region (*48*). A linear fit to the ensemble median indicates a −5 ± 4% per decade trend from 2003 to 2023 (Fig. 3J), corresponding to a net decline of −10 ± 8%. About 90% of ensemble members exhibit negative trends, indicating high confidence in a 21st century weakening of North African dust activity.

The associated dust loading over North Africa is 9.0 to 11.0 Tg (Fig. 4J), with emissions of ∼1800 to 2250 Tg year^−1^ (fig. S13, A to C) and deposition of ∼1650 to 1970 Tg year^−1^ (fig. S14, A to C). Within the region, the Southern Sahara and Sahel hold the largest share of dust loading (4.5 to 6.0 Tg) and shows a decline of −5 ± 8% per decade (Fig. 4C), while West and East North Africa also show stronger negative trends. As expected, these results confirm North Africa as the dominant contributor to the global dust budget.

Over Asia (Middle East and Central Asia and East Asia), the satellite ensemble shows DAOD of ∼0.09 to 0.16, with LIVAS showing a higher DAOD consistent with the Ridley *et al.* (*32*) constraints (Fig. 3, D and E). The inverse model, which is calibrated to the Ridley *et al.* (*32*) regional DAOD, tracks LIVAS, with DAOD between ∼0.125 and 0.175, peaking around 2009 and declining to ∼0.13 by 2023 (Fig. 3K). The aggregated Asian trend is −5 ± 6% per decade, indicating a modest but statistically uncertain decline (Fig. 3K). Regionally, both Middle East and Central Asia and East Asia show similar ∼−5 ± 10% per decade trends with substantial interannual variability (Fig. 3, D and E), consistent with previous studies that identify declining dust in parts of West and South Asia and East Asia driven by circulation and land-surface changes (*17*, *42*, *49*–*52*).

The inverse model indicates an Asian dust loading of 7.5 to 10.5 Tg (Fig. 4K), dominated by Middle East and Central Asia (5.5 to 8.1 Tg; Fig. 4D), with East Asia contributing 1.6 to 2.1 Tg (Fig. 4E). Emission and deposition fluxes over Asia range between ∼1700 and 2200 Tg year^−1^ (fig. S13K) and ∼1700 and 2050 Tg year^−1^ (fig. S14K), respectively, with trends of about −5% per decade. Together, North Africa and Asia account for ∼80 to 90% of global dust loading and thus drive most of the global dust decline.

In North America, Australia, South America, and Southern Africa, dust does not dominate total AOD, and satellite dust constraints are weaker. Here, we rely on MERRA-2, CAMS, and NAAPS to constrain the inverse model (Materials and Methods). The three reanalyses differ substantially in mean DAOD (0.005 to 0.03), reflecting low dust burdens and large relative uncertainties (Fig. 3, F to I). The ensemble reconstruction yields DAOD ranges of 0.010 to 0.022 over North America, 0.012 to 0.015 over Australia, and 0.010 to 0.015 over South America and Southern Africa (solid black line in Fig. 3, F to I). The ensemble envelopes generally encompass the spread of the individual reanalyses and closely track MERRA-2, which is intermediate between CAMS and NAAPS.

Linear DAOD trends in these secondary regions are small and not statistically robust: −8 ± 12% per decade over North America, −9 ± 9% per decade over Australia, +1 ± 11% per decade over South America, and −2 ± 11% per decade over Southern Africa (Fig. 3, F to I). Ensemble-mean dust loading remains within 0.35 to 0.9 Tg over the study period (Fig. 4, F to I), with trends similar in sign and magnitude to DAOD and not significantly different from zero. Emission and deposition budgets also show trends typically between −10 and +3% per decade with uncertainties of order 15 to 20% (figs. S13, F to I, and S14, F to I), indicating no robust changes in dust sources or sinks in these regions.

**Fig. 5.**
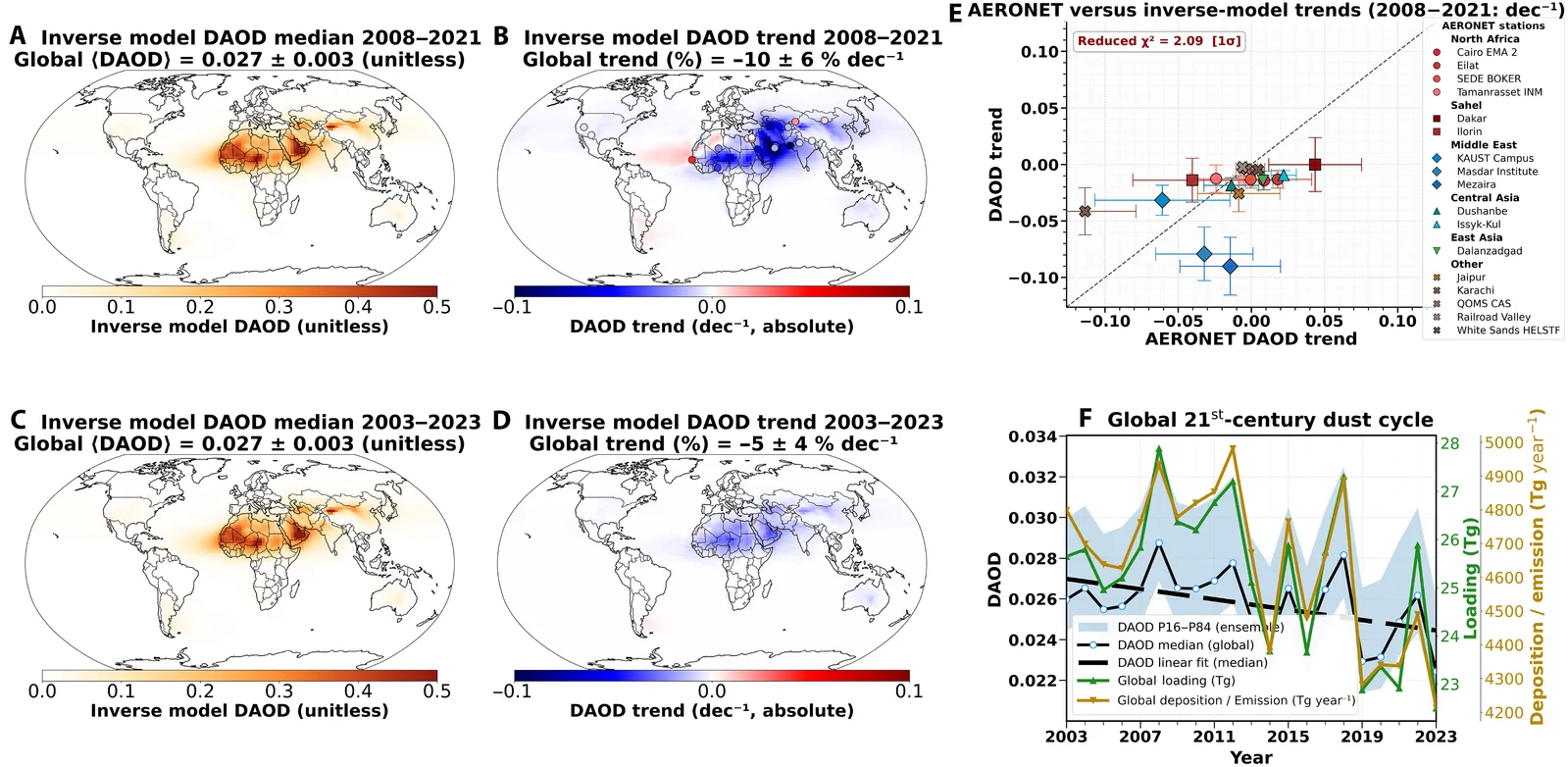
Inverse model reconstruction of global dust cycle attributes (2003–2023). (**A**) Multiyear (2008–2021) mean DAOD from the inverse model ensemble median. (**B**) Linear DAOD trend (2008–2021) expressed as percent per decade, computed at each grid cell from anomalies relative to that cell’s climatology over the analysis period. (**C** and **D**) Multiyear (2003–2023) mean DAOD and its linear trend from the ensemble median. (**E**) Comparison of decadal inverse model DAOD trends (2008–2021) with coarse-mode AOD trends derived at 16 dust-dominated AERONET sites (with reduced χ^2^ ≈ 2). (**F**) Time series of global dust cycle attributes: Symbols and shading show the ensemble-median with its one standard error (16th to 84th percentile) uncertainty range. Overlaid on the right-hand axes are global totals for global deposition and emission fluxes (gold, Tg year^−1^), and atmospheric loading (green, Tg).

Global-mean DAOD derived from the four satellite products ranges from 0.029 to 0.031 over 2003–2023 (Fig. 1, A, C, E, and G). The inverse model reconstructs similar values, 0.024 to 0.027 (Fig. 5, C and F), with slightly lower global means consistent with a modest underestimate of dust lifetime and long-range transport in the underlying models (*44*, *53*–*54*). Global DAOD exhibits substantial interannual variability over 2003–2023, with the highest values during 2007–2008 and 2017, and notably low values in 2014, 2019, and 2023. Despite this variability, a linear trend analysis indicates a −5 ± 4% per decade global DAOD trend (Fig. 5D), with similar declines in global dust loading (−10 ± 12% from 2003 to 2023) and in emission (fig. S13L) and deposition (fig. S14L) fluxes. More than 90% of ensemble members show a negative global DAOD change, indicating a robust weakening of the global dust cycle. Extending the MERRA-2 record through 2024 yields a comparable negative trend (−4 ± 2% per decade; fig. S15).

**Fig. 6.**
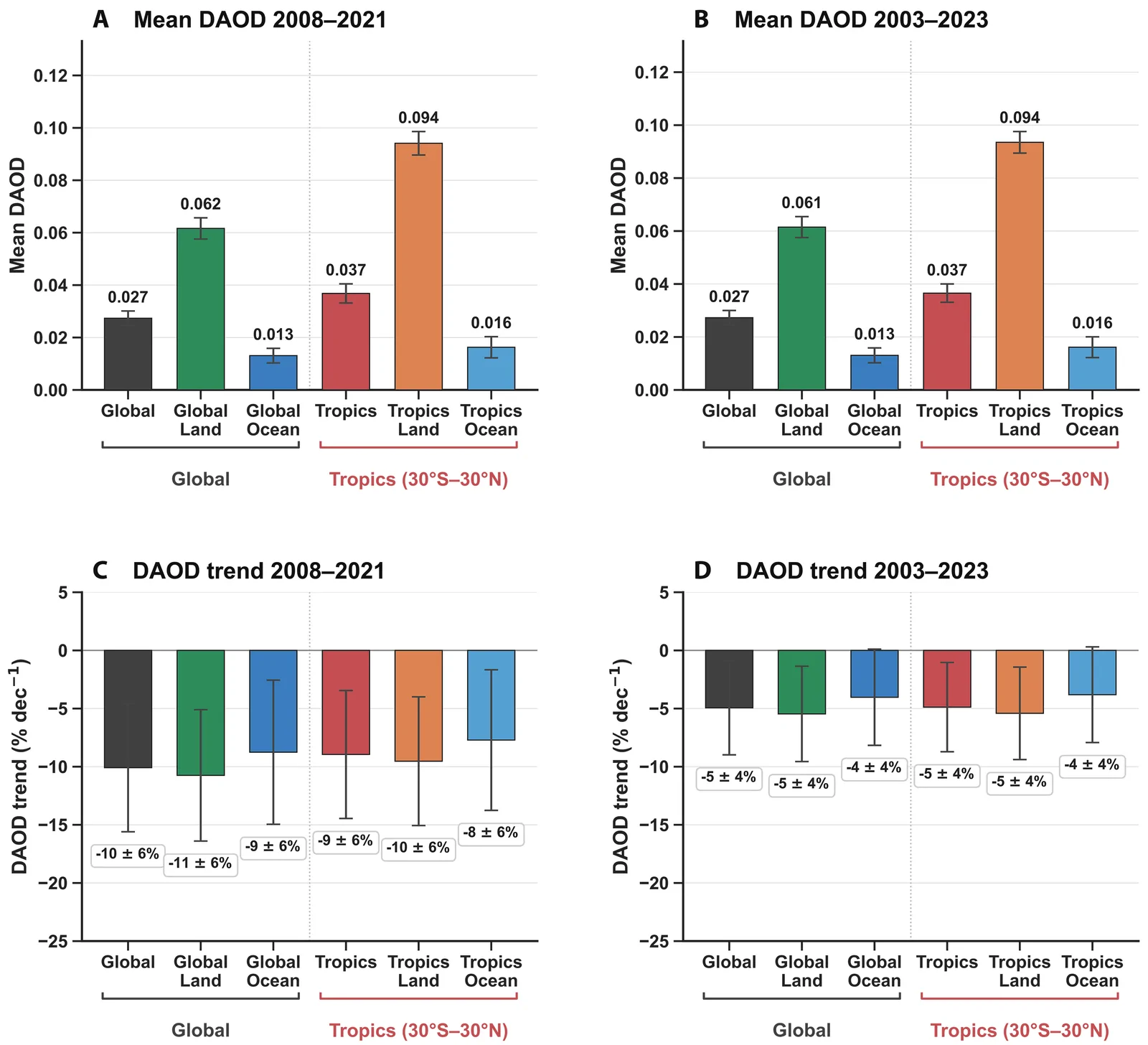
Domain decomposition of DAOD and its trends. Mean DAOD and decadal percentage trends are shown for global and tropical domains, separated into all-surface, land, and ocean regions, for 2008–2021 and 2003–2023. (**A**) Mean DAOD for 2008–2021. (**B**) Mean DAOD for 2003–2023. (**C**) Decadal percentage trends in DAOD for 2008–2021. (**D**) Decadal percentage trends in DAOD for 2003–2023. Bars indicate ensemble medians and error bars denote the 16th to 84th percentile range across ensemble members. Tropical regions are defined as 30°S to 30°N.

The global mean DAOD trend reflects partial cancellation between declining dust over North Africa and the Middle East and increasing dust over the North Atlantic. Decomposing the trend into land and ocean components (Fig. 6) reveals a stronger global decline over land (−11 ± 6% per decade) than ocean (−9 ± 6% per decade) for 2008–2021, with the same contrast persisting over 2003–2023 (−5 ± 4 versus −4 ± 4% per decade). Within the tropics (30°S to 30°N), the same pattern holds: Tropical land shows stronger declining trends (−10 ± 6% per decade for 2008–2021; −5 ± 4% per decade for 2003–2023) than tropical ocean (−8 ± 6%; −4 ± 4%). That both estimates overlap within their uncertainties indicates a global decline rather than a land-only phenomenon. The stronger continental signal is particularly relevant because land-surface energy budgets are more sensitive to dust radiative perturbations and more closely coupled to the warming in inhabited land regions.

### Possible causes of dust decline

Multiple past observational analyses show pronounced multidecadal variability in atmospheric dust during the satellite era, with global-mean DAOD rising in the early 2000s and declining after ∼2010 (*17*, *42*, *49*–*52*). Our observation-constrained reconstruction consolidates these signals into a coherent basis for the magnitude, timing, and spatial structure of recent dust variability.

A range of mechanisms has been proposed to explain post-2010 declines in dust in various regions, including weaker near-surface winds partially driven by a decreased meridional temperature gradient, land-surface changes (greening and increased soil moisture), and circulation changes that alter emission and transport efficiency, with additional modulation by region-specific hydroclimate variability and land management (*13*, *42*, *47*, *52*, *55*–*57*). However, robust attribution remains challenging because observational constraints on the dust cycle are still incomplete and, in some regions, inconsistent across sources, transport corridors, and sinks. We therefore focus on observational closure rather than attribution: By sharpening constraints on when global-mean DAOD declined and where the dominant spatial contributions arise, our reconstruction provides a stronger empirical foundation for targeted attribution studies of the post-2010 dust decline.

### Implications of the dust decline

The 10 ± 8% decline in atmospheric dust since the early 2000s has implications for Earth’s radiative balance and recent climate evolution. To estimate the radiative impact of this global dust decline, we use the recent estimate of the total present-day effective radiative effect (ERE) of dust of −0.2 ± 0.5 W m^−2^, which integrates dust interactions with radiation, clouds, chemistry, the cryosphere, and biogeochemistry (*3*).

Assuming, as a first-order approximation, that dust ERF (*58*, *59*) scales linearly with changes in global dust loading, an ∼10% reduction in dust burden implies a positive dust ERF of 10% of the dust ERE, equaling +0.02 ± 0.05 W m^−2^ over 2003–2023. This estimate should be interpreted cautiously, however, because (i) the increase of radiative effects with dust loading is usually less than linear, especially for interactions with clouds (*3*), and (ii) changes since the historical period in cloud cover, atmospheric temperature and humidity, surface properties, and the horizontal and vertical distribution of dust can also affect dust radiative effects, particularly in the longwave (*60*), even without changing global dust loading. This suggests that decreasing dust contributed modestly to both the growth of Earth’s energy imbalance (*8*) and to recent warming (*60*), including in 2023 and 2024, when record-high global temperatures coincided with anomalously low dust loading (Figs. 3, 4, and 5F and fig. S15) (*9*). This heating from reduced dust cooling occurred together with evidence that anthropogenic aerosol ERF has also become less negative since ∼2000 (*7*, *28*, *29*, *61*), suggesting a weakening of both natural and anthropogenic aerosol cooling that may have amplified recent warming.

Decreased dust loading also affects Earth’s planetary albedo. Dust scattering and absorption reduce absorbed solar radiation by ∼0.4 ± 0.2 W m^−2^ globally (one standard error range) (*3*); an ∼10% reduction in dust burden therefore produces a modest increase in absorbed solar radiation. Because dust loading is strongly hemispherically asymmetric, this reduction likely contributed slightly to the emerging hemispheric asymmetry in planetary albedo, with the Northern Hemisphere becoming relatively darker than the Southern Hemisphere (*62*).

Beyond climate and radiation, a weakening global dust cycle affects biogeochemical and ecological systems. A reduction in dust supply can alter iron fertilization of the open ocean, nutrient delivery to terrestrial ecosystems, and mineral deposition to cryosphere surfaces, with each process modulating carbon uptake and surface albedo (*3*, *63*). Moreover, reduced dust loading improves local and regional air quality, particularly in heavily dust-exposed areas (*64*). Furthermore, dust enhances snow and ice melt (*65*) and is the primary atmospheric source of iron and phosphorus to the open ocean, fertilizing phytoplankton productivity and modulating the biological carbon pump (*63*, *66*, *67*). A sustained dust decline could thus weaken the oceanic carbon sink in iron-limited regions (*66*), diminish the trans-Atlantic phosphorus supply to the Amazon basin (*67*), and reduce the snow-darkening effect that accelerates glacier melt (*65*).

In conclusion, seven different DAOD datasets and a 1000-member observation-constrained inverse model over the early 21st century indicate that global atmospheric dust has declined by ∼5 ± 4% per decade from 2003 to 2023. This dust decrease has influenced Earth’s energy balance, climate, and multiple components of the Earth system. By providing a continuous, observation-based record of natural aerosol variability, our reconstruction enables quantitative attribution of dust-driven climate forcing. If the observed linear trend was sustained, this modest decline would compound to a 15 to 30% reduction in global dust loading by midcentury, with implications for radiative forcing, ocean biogeochemistry and terrestrial nutrient supply, air quality, and other components of the Earth system. This extrapolation is illustrative rather than predictive, but underscores the need for continued observation and attribution of changes in the global dust cycle. These results provide a vital basis for incorporating the effects of the early 21st century dust decrease into climate model simulations, enabling investigations of recent warming, aerosol radiative forcing, and climate sensitivity that account for the effects of dust variability (*68*, *69*).

## MATERIALS AND METHODS

### Overview

To reconstruct changes in the global dust cycle over 2003–2023, we combined multiple satellite-derived DAOD datasets [LIVAS (*13*), MIDAS-Aqua (*16*), MIDAS-Terra (*16*), and IASI-LMD (*19*, *20*)], three aerosol reanalyses [MERRA-2 (*21*), CAMS (*22*), and NAAPS (*23*)], and an extended version of the DustCOMM inverse modeling framework (*44*, *45*). After analyzing these seven time series of DAOD (Figs. 1 and 3 and fig. S16) and evaluating their long-term trends against AERONET data (Fig. 2), we used inverse modeling to reconstruct the temporal evolution of the entire global dust cycle (e.g., DAOD, loading, and emission and deposition fluxes). To do so, we calibrated the four satellite DAOD products to the regional “ground truth” DAOD constraints of Ridley *et al.* (*32*) during their period of overlap (2004–2008). These calibrated satellite records, supplemented by reanalysis-based estimates in four minor dusty regions where satellite-derived DAOD is unreliable, are then ingested into the DustCOMM inverse model (*44*, *45*), which uses these constraints to optimally estimate source- and size-resolved dust emissions, transport, loading, and deposition for subsequent ∼5-year periods. Last, MERRA-2 is used to account for interannual variability within each ∼5-year period. This approach yields spatially and seasonally resolved, uncertainty-quantified reconstructions of the global dust cycle across the early 21st century (Figs. 3 to 5).

### Description of datasets used

To quantify changes in atmospheric dust over 2003–2023, we used DAOD products from satellite observations and aerosol reanalyses. Satellite DAOD was used to (i) assess regional and global DAOD variability and trends (figs. S1 to S3; see table S1) and (ii) construct monthly DAOD time series for 11 dust-dominant regions (boxes 1 to 11 in figs. S1 and S16), where dust contributes most of total AOD and satellite-based DAOD products are most reliable. These regional time series were used to constrain the DustCOMM inverse model (see below), which reconstructs the global dust cycle (DAOD, loading, emission, and deposition) over 2003–2023.

### DAOD datasets and sensor considerations

For the dust-dominant regions, four satellite-based DAOD datasets, MIDAS-Terra and MIDAS-Aqua (*16*, *70*), LIVAS (*13*, *71*), and IASI-LMD (*19*, *20*, *72*–*74*), were harmonized to a common 0.5° by 0.5° grid and monthly means (2008–2021) for intercomparison and evaluation (see table S1).

MIDAS derives DAOD by applying the dust–to–total AOD ratio from MERRA-2 to MODIS-retrieved total AOD (*16*). In MERRA-2, only the total, species-integrated AOD is assimilated from observations (*21*); the dust fraction itself comes from the GEOS-5/GOCART simulation and is not directly constrained by observations. Biases in the simulated dust life cycle, such as emission, transport, and deposition, can therefore propagate into the MIDAS DAOD estimates. That said, Gkikas *et al.* (*16*) showed that the MERRA-2 dust–to–total AOD ratio agrees well with the LIVAS dust fraction, particularly over dust-rich regions (their figures 1, 2, S2, and S4), and that MIDAS DAOD compares well with AERONET-based DAOD (their sections 2.4 and 4.2).

Sampling time differs across sensors and can influence apparent trends via diurnal variability and event timing. MIDAS Terra and Aqua have nominal equator-crossing times of ∼10:30 (Terra) and ∼13:30 (Aqua) local solar time, respectively. CALIOP/CALIPSO (basis of LIVAS) flies in the A-Train with equator crossings near ∼13:30 and ∼01:30 local solar time. IASI aboard MetOp provides two daily overpasses with approximate local equator-crossing times of ∼09:30 (descending) and ∼21:30 (ascending). These sampling differences should be considered when interpreting interdataset discrepancies in DAOD levels and trends; we therefore use AERONET as an independent, ground-based reference for assessing the robustness of the inferred trends.

For AERONET (*43*, *75*) trend comparisons (Fig. 2), we averaged the four 0.5° cells within ±0.5° of each site, applied the selected three-criterion (3C) filter: a coarse-mode AOD proxy; dust–to–sea salt ratio > 3; and ≥8 valid months year^−1^ for ≥6 years. We then computed monthly anomalies relative to the 2008–2021 climatology and estimated linear trends (decade^−1^) by least squares regression with standard-error uncertainties. Sensitivity tests around our selected 3C thresholds indicate that the inferred mean AERONET trend remains negative across reasonable filter choices, with no trend sign reversal (fig. S17).

As stated above, for regions where dust is important but not typically dominant (North America, South America, Southern Africa, and Australia), satellite dust isolation is less robust (*47*); for these, we used DAOD from three reanalyses: MERRA-2 (*21*), CAMS (*22*), and NAAPS-RA (*23*).

Because products rely on different sensors and assumptions, we considered key factors that can affect the estimated trends: (i) dust detection approach and size sensitivity, with CALIOP/LIVAS using depolarization-based dust typing (*13*), IASI detecting dust in the thermal infrared and applying an infrared-to-visible mapping (*19*, *20*, *72*), and MODIS/MIDAS deriving dust by applying a reanalysis-based dust fraction to total AOD (*16*, *21*); (ii) sampling differences (above, and along-track versus swath sampling); and (iii) retrieval limitations, including bright-surface/cloud impacts for passive sensors, CALIOP sensitivity limits for optically thin layers and higher daytime noise (*71*), and reanalysis DAOD dependence on emissions/deposition and aerosol partitioning given assimilation of total AOD (*21*, *22*). We mitigate these effects by focusing satellite constraints on dust-dominant regions, applying consistent gridding/aggregation, and evaluating trends against AERONET and across different products (*32*, *76*–*77*) (see below).

We retained IASI-LMD v2.2 only over major desert dust-source regions, the Sahara (0° to 40°N, 20°W to 30°E) and Asian dust regions (10° to 50°N, 60° to 140°E), where its performance is most robust (*74*). Outside these regions, IASI-LMD was fused with LIVAS dust AOD using spatial weighting masks. The LIVAS-fused IASI field was calculated as

$${\text{DAOD}}_{\text{LIVAS}-\text{fused IASI}}=w\;{\text{DAOD}}_{\text{IASI}-\text{LMD}}+\left(1-w\right)\;{\text{DAOD}}_{\text{LIVAS}}$$ (1)

where w is a latitude-longitude weight that decreases gradually away from the two retained source regions. Separate weights were computed for the Sahara and Asian source regions, each close to one within its region and decreasing smoothly toward zero away from it over a 5° latitude-longitude smoothing scale. This smooth decay avoids an abrupt transition between IASI-LMD and LIVAS at the source region boundaries. At each grid point, “*w*” was set to the larger of these two regional weights. The resulting LIVAS-fused IASI dataset therefore preserves IASI-LMD information near major dust sources and increasingly relies on LIVAS farther away. Unless otherwise stated, we used the LIVAS-fused IASI dataset in place of native IASI-LMD throughout the analysis.

We obtain the Copernicus C3S IASI-LMD v2.2 dust optical depth product (LMD AERO v2.2 CDR), available from the ICARE/C3S archive (www.icare.univ-lille.fr/asd-content/archive/?dir=C3S-Aerosols/IASI_LMD_V2.2). IASI-LMD retrieves dust optical depth in the thermal infrared at 10 μm, and DAOD at 550 nm (*78*) is a visible-equivalent estimate obtained via Lorenz-Mie–based infrared-to-visible conversion ratios (*20*): 0.85 for the Balkanski *et al.* (*79*) refractive index and 0.56 for Volz *et al.* (*80*). Capelle *et al.* (*78*) (ATBD) notes that these uniform ratios are approximate, since the true ratio depends on dust size distribution, mineralogy, and refractive index, which vary regionally and may drift on decadal timescales.

In our inverse modeling framework, all satellite products ingested into DustCOMM, including IASI-LMD, are calibrated to Ridley *et al.* (*32*) over 2004–2008 on a regional basis; source-dependent biases from the uniform conversion are therefore absorbed into region-specific calibration factors, and any time-invariant component of the conversion bias does not propagate to our calibrated DAOD, dust loading, emissions, deposition, or trend estimates. Residual uncertainty in IASI-derived trends arises only if the true infrared-to-visible conversion ratio itself drifts over time within a region, for instance, through long-term changes in dust size distribution, mineralogy, or source-region mixture.

### Selection of dust-dominated AERONET stations for DAOD trend evaluation

We assess whether the four satellite-based and three reanalysis DAOD products can capture long-term trends in DAOD by comparing them with ground-based AERONET observations. We use daily-averaged, Level 2.0 quality-assured coarse-mode AOD from AERONET version 2. Daily values are aggregated to monthly means for 2008–2021, and dust-scene filters (see below) are applied so that coarse-mode AOD primarily represents mineral dust (*16*).

Coarse-mode AOD is a reliable DAOD proxy at Saharan and near-Saharan sites, where dust dominates the coarse mode (*81*–*83*). In coastal and marine-influenced regions, however, sea salt and other nondust coarse particles also contribute to coarse-mode AOD, so the coarse-mode AOD proxy can overestimate DAOD. To avoid such contamination, we do not attempt to disentangle dust from sea salt at the AERONET level; instead, we apply an independent MERRA-2 dust–to–sea salt ratio filter (3C-filtered) to exclude sites where nondust coarse aerosols are likely to dominate the coarse-mode signal (*84*).

Because total AOD can include substantial fine-mode contributions from pollution and biomass burning, we applied a study-specific screening procedure to identify AERONET sites with predominantly dust-related coarse-mode variability. The procedure was motivated by previous dust-attribution studies (*85*, *86*), but the exact threshold combination is defined for this analysis. To select dust-dominated AERONET stations, we retain a station if (i) monthly mean coarse-mode AOD accounted for ≥35% of total AOD; (ii) the record included ≥6 years with ≥8 valid monthly means per year during 2008–2021; and (iii) the colocated 0.5° by 0.5° MERRA-2 grid cell had an annual mean DAOD–to–sea salt AOD ratio > 3, to reduce marine-aerosol influence (*86*). Stations satisfying all three criteria are termed “3C-filtered AERONET stations.” Sixteen of the 1440 stations satisfy these requirements. For each station, we build a 2008–2021 month-of-year coarse-mode AOD climatology, remove it to obtain deseasonalized anomalies, and estimate decadal trends using ordinary least squares.

We assessed the robustness of the AERONET-derived trends to the station-selection thresholds using sensitivity tests (fig. S16). Each criterion was varied independently while the other two were fixed at their reference values, spanning coarse-mode AOD fraction thresholds of 20 to 50%, DAOD–to–sea salt AOD ratio thresholds of 1.5 to 5.0, and minimum data-coverage requirements of 4 to 10 years. We also jointly varied the coarse-mode AOD fraction and DAOD–to–sea salt AOD ratio thresholds to test interactions between the two aerosol-composition criteria, while retaining the reference minimum-years requirement. Across these tests, the mean AERONET trend remained consistently negative across the tested threshold combinations (fig. S16).

### Validation and trend intercomparison with AERONET stations

To evaluate how closely each DAOD dataset reproduces observed dust trends, we compared station-by-station decadal trends against the 3C-filtered AERONET coarse-mode AOD records. Because the emphasis is on long-term change rather than instantaneous retrieval agreement, we analyzed monthly-mean, deseasonalized anomalies without strict temporal collocation. Exact temporal collocation between AERONET data and the other DAOD products is not imposed, but the focus on monthly means, deseasonalization, and the requirement of ≥8 valid AERONET months per year reduce sensitivity to sampling differences; any remaining mismatches are expected to increase variance rather than systematically bias decadal trend estimates.

For each of the 16 AERONET sites and each of the seven DAOD products, we extracted monthly DAOD from the model/satellite grid cell whose center lies within ±0.5° of the station for all months with available observations. We then applied linear regression to the deseasonalized time series to estimate a decadal trend at each station, $\zeta_{i}^{\text{prod}}$, and derived the corresponding AERONET trend, $\zeta_{i}^{\text{AER}}$, from the coarse-mode AOD record. Agreement was quantified using the reduced chi-square statistic

$$\chi_{\text{red}}^{2}=\frac{1}{N}\sum_{i=1}^{N}\frac{{\left(\zeta_{i}^{\text{prod}}-\zeta_{i}^{\text{AER}}\right)}^{2}}{{\left(\sigma_{i}^{\text{prod}}\right)}^{2}+{\left(\sigma_{i}^{\text{AER}}\right)}^{2}}$$ (2)

where N is the number of stations and $\sigma_{i}^{\text{prod}}$ and $\sigma_{i}^{\text{AER}}$ are the standard errors of the regression slopes for the DAOD product and AERONET, respectively. Values of $\chi_{\text{red}}^{2}\approx 1$ indicate agreement within combined uncertainties, whereas $\chi_{\text{red}}^{2}>1$ and $\chi_{\text{red}}^{2}<1$ indicate larger- or smaller-than-expected differences, respectively.

For the main trend comparison, we retained the 16 annually qualified 3C-filtered stations and computed seasonal trends using this fixed ensemble, ensuring that seasonal differences reflect temporal variability rather than changes in station composition. As a sensitivity check, applying the 3C criteria separately by season yielded larger station sets, particularly in MAM and JJA (54 and 45 stations, respectively; fig. S18), including North Atlantic and Caribbean sites downstream of Saharan outflow. These seasonally qualified sites include positive boreal-summer trends at La Parguera and Ragged Point (+0.08 and +0.13 decade^−1^), consistent with the positive MODIS-family DAOD signal over the tropical North Atlantic. Cape Verde does not meet the 3C criteria even seasonally.

### DustCOMM inverse model methodology

The DustCOMM inverse modeling framework (*44*–*45*) constrains the global dust cycle by combining observations with an ensemble of simulations from six global dust models: CESM (*87*), IMPACT (*88*), GISS Model (*89*), GOCART (*90*), MONARCH (*91*–*92*), and INCA (*93*). Each model performed source-tagged simulations for nine major low-latitude dust source regions (fig. S1), resolving particle sizes up to 20 μm (*44*). Five-year mean simulations were used to reduce the influence of interannual meteorological variability on emission, transport, and deposition (*46*). The nine regions represent >99% of global low-latitude desert dust emissions (*44*).

DustCOMM combines an ensemble of global model simulations with observational constraints on dust properties and abundance through inverse modeling (*44*, *45*, *47*, *59*). The full workflow, the model ensemble, dust size distribution, size-resolved extinction efficiency, inverse-model formulation, and bootstrap-based uncertainty propagation all follows Kok *et al.* (*44*) and is unchanged here. The only new adaptation in this study is the DAOD constraint. Instead of the single climatological constraint from Ridley *et al.* (*32*), we use period-specific regional DAOD values for each of the four 5-year windows, derived from our multiproduct observational ensemble (MIDAS-Aqua, MIDAS-Terra, IASI-LMD, and LIVAS) and applied across the 15 source and near-source regions in (*44*). This change lets the inverse model produce time-resolved reconstructions of dust optical depth, loading, emissions, and deposition.

The DustCOMM methodology uses optimal estimation to infer the optimal contributions of each source region and size bin that minimizes disagreement with observational constraints on dust size distributions, extinction efficiencies and regional DAOD across 15 source and near-source regions (*32*, *47*). The resulting product provides global gridded dust loading, DAOD, concentrations, and emission/deposition fluxes by season, source region, and particle size, with uncertainties estimated via bootstrap propagation of model spread and uncertainties in dust microphysical properties and DAOD constraints (*59*, *94*). The resulting 5-year climatology better represents the Northern Hemisphere dust cycle relative to climate/chemical transport models and MERRA-2, with a more modest improvement in the Southern Hemisphere dust cycle (*44*, *95*). We further show seasonal global DAOD distributions and trends from the inverse-model ensemble in fig. S19, indicating that the negative annual-mean trend is dominated by negative trends during spring, summer, and autumn.

### Regional DAOD constraints for inverse modeling of 21st century dust cycle

Previously, the DustCOMM inverse modeling methodology was used to obtain the global dust cycle for the period 2004–2008. Here, we extend that work by obtaining constraints on the global dust cycle for the successive 5-year periods of 2003–2008, 2009–2013, 2014–2018, and 2019–2023. To do so, we require seasonal DAOD constraints for the 15 dusty regions (see figs. S1 and S15) in each 5-year interval.

Observational DAOD data come from three primary sources: (i) Ground-based observations, such as from AERONET, provide a direct measurement of AOD with high accuracy; however, AERONET has limited spatial coverage and can only crudely partition optical depth into fine and coarse aerosol contributions. (ii) Satellite-based observations, such as from MODIS-Aqua and MODIS-Terra, CALIPSO, and IASI, offer near-global coverage but cannot accurately separate dust from other aerosol types. Additionally, satellite sensors cannot directly measure the AOD, and instead, they rely on retrieval algorithms to infer AOD at different wavelengths, which introduces higher uncertainty and possible biases compared with ground-based (AERONET) data. (iii) Global atmospheric models provide global coverage and can distinguish DAOD from AOD from other aerosol components, but their fidelity is lower than that of both ground- and satellite-based AOD datasets.

Ridley *et al.* (*32*) leveraged the complementary strengths of these three different data sources (ground-based observations, satellite observations, and global model results) to constrain the seasonal DAOD in 11 regions where dust dominates the AOD (see red rectangles in fig. S1). Specifically, they used AERONET data to calibrate and bias-correct satellite-based AOD data in these dusty regions and subsequently applied an ensemble of model results to remove the nondust contribution of the AOD, yielding DAOD estimated for the period 2004–2008. We therefore consider the Ridley *et al.* (*32*) product to be the most accurate regional DAOD dataset currently available, and we use it as the reference dataset for calibrating the satellite-derived DAOD products over their overlap period (2004–2008). The calibrated satellite products, not the Ridley *et al.* (*32*) dataset itself, provide the DAOD constraints for all subsequent 5-year periods for the full 2003–2023 record.

### Calibration of MIDAS-Aqua, MIDAS-Terra, LIVAS, and IASI DAOD

To ensure consistency across observational datasets, we calibrated the satellite-derived DAOD products [LIVAS (*13*), MIDAS-Aqua (*16*), MIDAS-Terra (*16*), and IASI-LMD (*19*–*20*)] against the regional DAOD constraints from Ridley *et al.* (*32*), which provide the most accurate available reference for dusty regions. Because satellite retrievals can exhibit systematic biases, arising from surface reflectance effects, assumptions in aerosol optical properties, and instrument-specific degradation, we expect these products to deviate more than the adjusted Ridley *et al.* (*32*) regional DAOD estimates.

As stated above, we applied IASI-LMD only over major desert source regions, where its performance is most robust; in other regions, IASI gaps are supplemented using LIVAS dust AOD to create a LIVAS-fused IASI dataset (see Eq. 1). The extensive regional uncertainty characterization provided by Ridley *et al.* (*32*) enables systematic propagation of reference-product errors into the inverse model (*44*). By calibrating each satellite product to the Ridley *et al.* (*32*) climatology for 2004–2008, we implicitly assume that relative biases between the satellite products and the reference dataset remain temporally stable. Last, by using multiple, relatively independent satellite-based DAOD records, we exploit the spread in their inferred trends as an estimate of observational uncertainty (*96*).

The four dust products analyzed here differ both in their retrieval algorithms and in their sampling characteristics (*13*, *14*, *19*, *20*), which has important implications for interproduct consistency and for the common calibration applied in this study. To place the three products on a common scale, we apply a uniform calibration based on seasonal DOD averages from Ridley *et al.* (*32*). However, because each product samples the atmosphere differently in space and time, this “common” calibration implicitly assumes that the Ridley *et al.* (*32*) seasonal climatology is sampled equivalently by all instruments. In practice, mismatches in temporal sampling (overpass time and clear-sky conditions) and spatial sampling (coverage gaps and swath geometry) introduce additional uncertainty in the calibration factors and may lead to residual, sensor-specific systematic biases that we cannot fully quantify. We therefore treat these calibration-related uncertainties and sampling-driven biases as an intrinsic limitation of the intercomparison and revisit their implications when interpreting decadal trends with 1000 ensemble runs.

The MIDAS-Terra and MIDAS-Aqua satellite data products are available for the entire study period (2003–2023). However, LIVAS (2007–2022) and IASI (2008–2021) do not span the full 2003–2023 period, so missing regional seasonal DAOD values are inferred from MIDAS during the periods of overlap. For dusty region *r*, missing year *y* and season *s*, DAOD for product *k* (IASI or LIVAS) is estimated from product *m* (MIDAS-Terra or MIDAS-Aqua) as

$$\tau_{k}\left(r,s,y\right)=\gamma_{m}\left(r,s\right)\tau_{m}\left(r,s,y\right)$$ (3)

where $\tau_{k}$ is the area-weighted mean DAOD of satellite product *k*, and $\gamma_{m}\left(r,s\right)$ corrects for systematic differences between *k* and *m*. This correction factor is drawn from a normal distribution $N∼\left({\bar{\gamma }}_{m},\sigma_{\gamma_{m}}\right)$ (see discussion of bootstrap procedure below) for which the distribution mean ${\bar{\gamma }}_{m}$ equals

$${\bar{\gamma }}_{m}\left(r,s\right)=\frac{1}{N_{\text{ovl}}}\sum_{y=y_{\text{st},m}}^{y_{\text{end},m}}\frac{\tau_{k}\left(r,s,y\right)}{\tau_{m}\left(r,s,y\right)}$$ (4)

where year $y_{\text{st},m}$ equals 2007 for LIVAS and 2008 for IASI and year $y_{\text{end},m}$ equals 2022 for LIVAS and 2021 for IASI, yielding $N_{\text{ovl}}=\;16$ for LIVAS and $N_{\text{ovl}}=14$ for IASI. The uncertainty $\sigma_{\gamma_{m}}\left(r,s\right)$ is the standard deviation of the ratio $\frac{\tau_{k}\left(r,s,y\right)}{\tau_{m}\left(r,s,y\right)}$ across the $N_{\text{ovl}}$ overlapping years.

After filling the LIVAS and IASI records using Eqs. 3 and 4, we calibrated each satellite-based product *j* [LIVAS (*13*), MIDAS-Aqua (*16*), MIDAS-Terra (*16*), and IASI-LMD (*19*, *20*)] to the DAOD from Ridley *et al.* (*32*) using

$$\tilde{\tau }\left(r,s,y\right)=\beta_{j}\left(r,s\right)\tau_{j}\left(r,s,y\right)$$ (5)

where $\beta_{j}\left(r,s\right)$ is drawn from a normal distribution $N∼\left({\bar{\beta }}_{j},\sigma_{\beta_{j}}\right)$ with

$${\bar{\beta }}_{j}\left(r,s\right)=\frac{{\bar{\tau }}_{\text{ridl}}\left(r,s\right)}{{\bar{\tau }}_{j}\left(r,s\right)}$$ (6)

and overbars denote seasonal means averaged over the 2004–2008 period. ${\bar{\tau }}_{\text{ridl}}\left(r,s\right)$ is the constraint on the DAOD averaged over season *s*, region *r*, and years 2004–2008 as obtained by Ridley *et al.* (*32*). For MIDAS-Terra and MIDAS-Aqua, ${\bar{\tau }}_{j}\left(r,s\right)$ is directly obtained from all 5 years of data. For LIVAS, missing 2004–2006 DAOD values are estimated using Eq. 3. For IASI, missing 2004–2007 values are likewise estimated using MIDAS-based scaling.

Equations 4 and 5 thus yield product-specific calibration factors ${\bar{\beta }}_{j}$ that adjust each multiyear DAOD record to be consistent with the Ridley *et al.* (*32*) regional DAOD constraints. The error in this calibration, $\sigma_{\beta_{j}}$, equals the error $\sigma_{{\bar{\tau }}_{\text{ridl}}}\left(r,s\right)$, quantified in Ridley *et al.* (*32*), which accounts for multiple sources of uncertainty, including cloud-screening of the satellite (MODIS Terra, MODIS Aqua, and MISR) and ground-based (AERONET) data, as well as uncertainties in model-based removal of nondust AOD from calibrated satellite retrievals. As such, Ridley *et al.* (*32*) is used only to ground the absolute magnitude of each satellite product during 2004–2008; the calibrated satellite records provide the regional DAOD constraints for the full 2003–2023 time series.

### Time series of seasonal DAOD for the four minor dusty regions

The procedure above provides calibrated seasonal DAOD constraints for 11 dust-dominated regions. The DustCOMM inverse model additionally requires constraints for four “minor” dusty regions (North America, South America, South Africa, and Australia), where dust is important but does not dominate AOD (fig. S16). In these regions, the Ridley *et al.* (*32*) approach is not suitable because uncertainties in modeled nondust aerosols can translate into large, weakly constrained DAOD errors (*47*). We therefore estimate $\tilde{\tau }\left(r,s,y\right)$ as the area-weighted seasonal mean DAOD from three aerosol reanalyses [MERRA-2 (*21*), CAMS (*22*), and NAAPS (*23*)]. Because NAAPS does not provide 2023, we fill that year using MERRA-2 and CAMS following the same correction procedure used for missing LIVAS and IASI years.

### Error analysis using a bootstrap procedure

To propagate observational and calibration uncertainties, and to account for error correlations across years introduced by applying common calibration factors (e.g., Eq. 6), we generate an ensemble of seasonal DAOD constraints using bootstrap resampling:

1) Dataset selection: Randomly select one satellite product j (MIDAS-Terra, MIDAS-Aqua, LIVAS, or IASI) and one reanalysis k (CAMS, MERRA-2 or NAAPS).

2) Satellite data gap filling: If LIVAS or IASI is selected, fill missing years using Eqs. 2 and 3 with a randomly chosen MIDAS product m (Terra or Aqua), drawing $\gamma_{m}\left(r,s\right)$ from $N∼({\bar{\gamma }}_{m},\sigma_{\gamma_{m}})$ for each region/season/missing year.

3) Calibration (dust-dominated regions): For each of the 11 dust-dominated regions and each season, calibrate all years to Ridley *et al.* (*32*) by drawing $\beta_{j}\left(r,s\right)$ from $N∼({\bar{\beta }}_{j},\sigma_{\beta_{j}})$, yielding $\tilde{\tau }\left(r,s,y\right)$. Correlated regional/seasonal systematic errors are treated as in Kok *et al.* (*44*).

4) Minor regions: For the four minor regions, draw $\tilde{\tau }\left(r,s,y\right)$ from $N∼\left[\tau_{k}\left(r,s,y\right),\sigma_{\tau_{k}}\left(r,s,y\right)\right]$ using the selected reanalysis k and assuming 10% relative uncertainty [Ridley *et al.* (*32*)], with correlated errors handled as in Kok *et al.* (*44*). If NAAPS is selected, fill 2023 using MERRA-2 or CAMS.

5) Aggregation: Average seasonal DAOD within each ∼5-year period.

6) Inverse model runs: Use the resulting DAOD constraints for the 15 regions (see figs. S1 and S16) to drive the DustCOMM inverse model for each period (2003–2008, 2009–2013, 2014–2018, and 2019–2023) (*44*).

This yields an ensemble of global dust-cycle realizations for each ∼5-year period (including DAOD, loading, emissions, and deposition), resolved by season, particle size, source region, and space (*44*, *45*). Using multiple DAOD products in the ensemble allows the spread in product-specific DAOD trends to quantify observational uncertainty (*96*). The resulting ensembles of global dust cycle variables represent their probability distributions; we report the median and 16th to 84th percentile range as uncertainty (fig. S16). These ranges incorporate random and product-specific systematic errors in the inputs and correction factors, but do not capture biases shared across all DAOD datasets and therefore likely represent a lower bound on total uncertainty.

### Accounting for interannual variability within each ∼5-year climatology

Our reconstruction yields a sequence of ∼5-year dust-cycle climatologies. This assumes that meteorological conditions within each period are broadly comparable to those in 2004–2008, the period for which the source- and size-resolved global dust cycle simulations used in the DustCOMM inverse model were generated (*44*, *45*). To incorporate interannual variability within each ∼5-year climatology obtained by the inverse model, we scaled each inverse-model seasonal climatology using reanalysis data

$$x\left(\theta ,\phi ,s,y\right)={\bar{x}}_{\text{inv}}^{p}\left(\theta ,\phi ,s\right)\frac{x_{\text{rean}}\left(\theta ,\phi ,s,y\right)}{{\bar{x}}_{\text{rean}}^{p}\left(\theta ,\phi ,s\right)}$$ (7)

where x(θ,ϕ,s,y) denotes a dust property (e.g., DAOD, loading, emission, or deposition flux) at location (θ,ϕ), season s, and year y. Overbars denote averages over the ∼5-year period p containing year y; “inv” indicates the inverse-model climatology and “rean” the corresponding reanalysis field. We use MERRA-2 to define $x_{\text{rean}}$ and ${\bar{x}}_{\text{rean}}^{p}$ because it best reproduces observed long-term trends relative to AERONET (Fig. 2).
