# Supplementary material for: A global decline in atmospheric dust during the 21st century
Source: Sci Adv. 2026 Sep 16;12(38):eaef5691. doi: 10.1126/sciadv.aef5691 (PMC13580573; doi:10.1126/sciadv.aef5691)
Supplement: Supplementary file 1 [file sciadv.aef5691_sm.pdf]

Science Advances
AAAS

Supplementary Materials for

A global decline in atmospheric dust during the 21st century

Ashok Kumar Gupta *et al.*

Corresponding author: Ashok Kumar Gupta, ashok.k.gupta@vanderbilt.edu

Sci. Adv. **12**, eaef5691 (2026)
DOI: 10.1126/sciadv.aef5691

This PDF file includes:

Figs. S1 to S19
Tables S1 to S2

Supplementary Figures

Figure S1

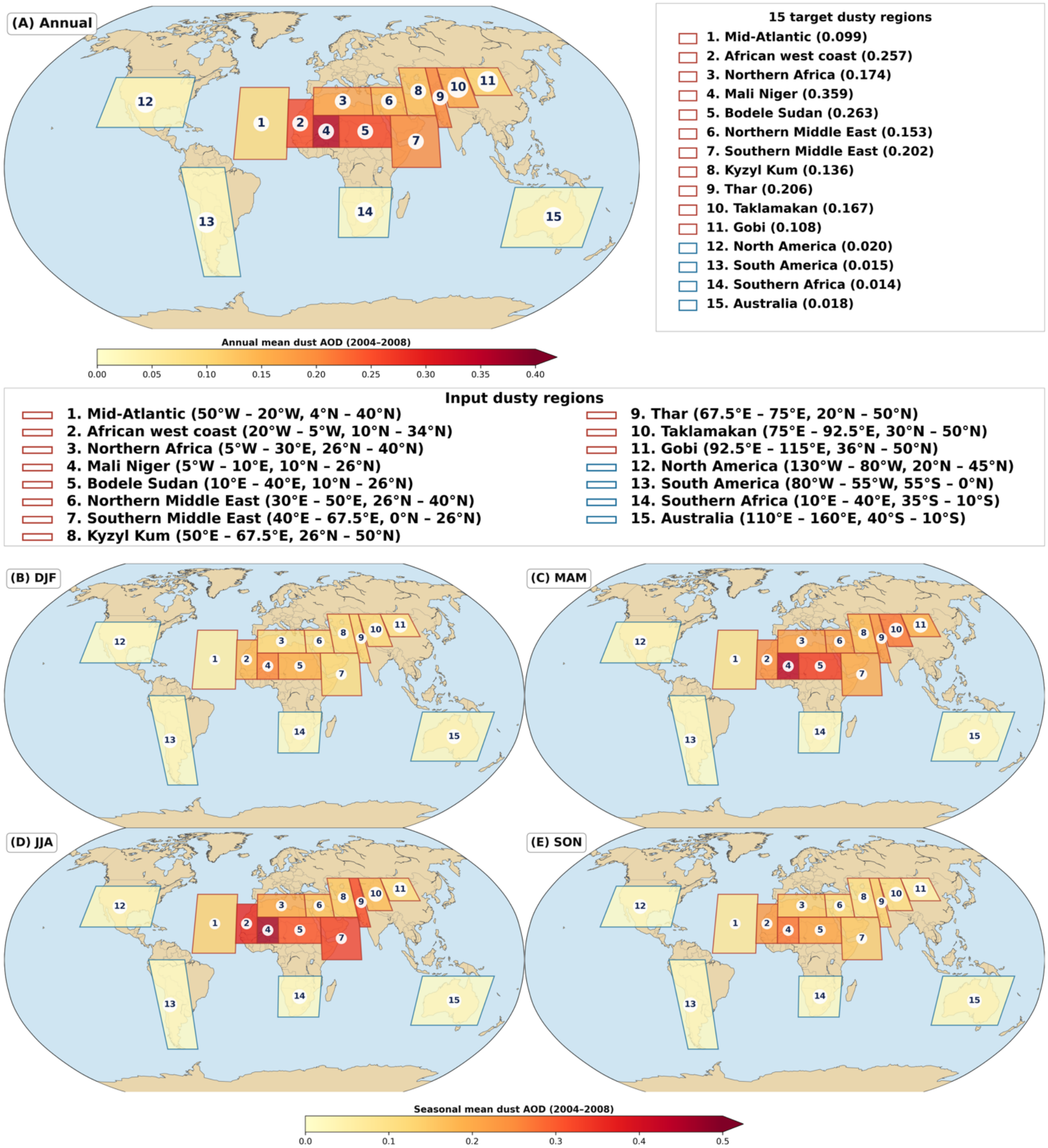

Seasonal mean DAOD by region

Region	DJF	MAM	JJA	SON
1. Mid-Atlantic	0.064	0.106	0.143	0.084
2. African west coast	0.180	0.250	0.365	0.233
3. Northern Africa	0.118	0.219	0.207	0.151
4. Mali Niger	0.257	0.441	0.462	0.277
5. Bodele Sudan	0.191	0.339	0.310	0.212
6. Northern Middle East	0.112	0.223	0.164	0.113
7. Southern Middle East	0.123	0.204	0.330	0.150
8. Kyzyl Kum	0.115	0.176	0.154	0.101
9. Thar	0.130	0.238	0.319	0.135
10. Taklamakan	0.119	0.275	0.171	0.104
11. Gobi	0.093	0.192	0.102	0.047
12. North America	0.010	0.029	0.028	0.012
13. South America	0.019	0.013	0.010	0.016
14. Southern Africa	0.016	0.011	0.013	0.016
15. Australia	0.025	0.013	0.010	0.023

Figure S1: *Global distribution of the 15 dusty regions used by the inverse model, and their annual and seasonal mean DAOD over 2004–2008.* ***(A)*** *Annual mean DAOD;* ***(B–E)*** *seasonal*

*mean DAOD for **(B)** DJF, **(C)** MAM, **(D)** JJA, and **(E)** SON. Spatial boundaries of the 15 dusty regions for which the regionally averaged DAOD is used in the inverse model (Kok et al (44)) were delineated based on satellite-derived dust climatologies and prior observational and modeling studies (Ridley et al (32)). Red boxes denote regions where dust generally dominates the AOD, while blue boxes indicate less dusty regions where dust does not dominate the AOD. The numbered regions correspond to those listed in the accompanying legend, including major dust hotspots such as the Sahara (Regions 3–5), the Middle East (Regions 6–7), Central and East Asia (Regions 8–11), and lesser sources in the Americas, Australia, and southern Africa (Regions 12–15). Region-resolved seasonal values are tabulated below the maps, and the Ridley annual mean DAOD for each region is shown in parentheses next to the region name in the legend. The 16 AERONET stations used for trend comparisons are listed in table S2.*

Figure S2

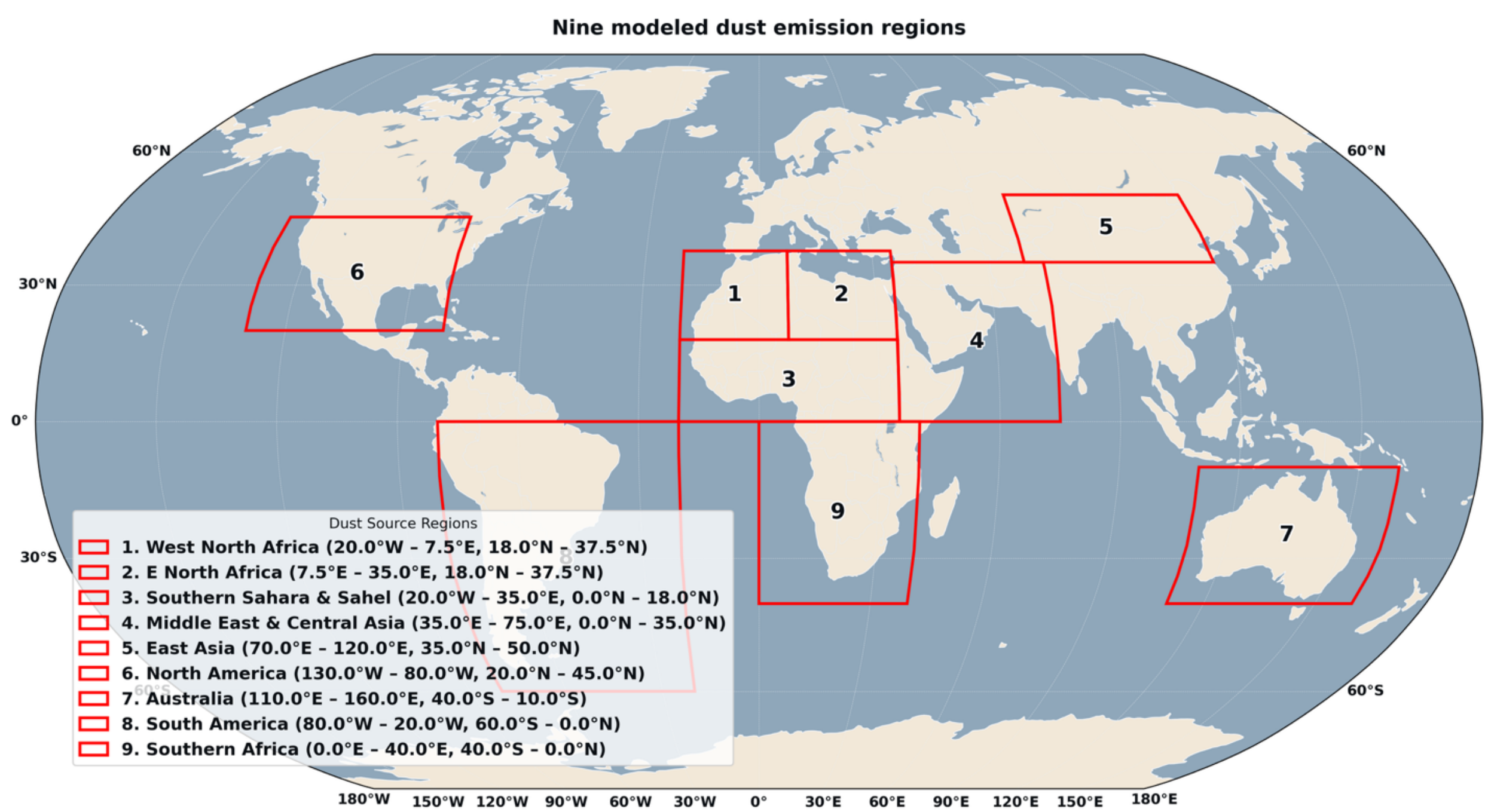

Figure S2: Global distribution of 9 modeled dust emission source regions. Map showing the global locations of the nine principal dust emission source regions identified in the study.

Figure S3

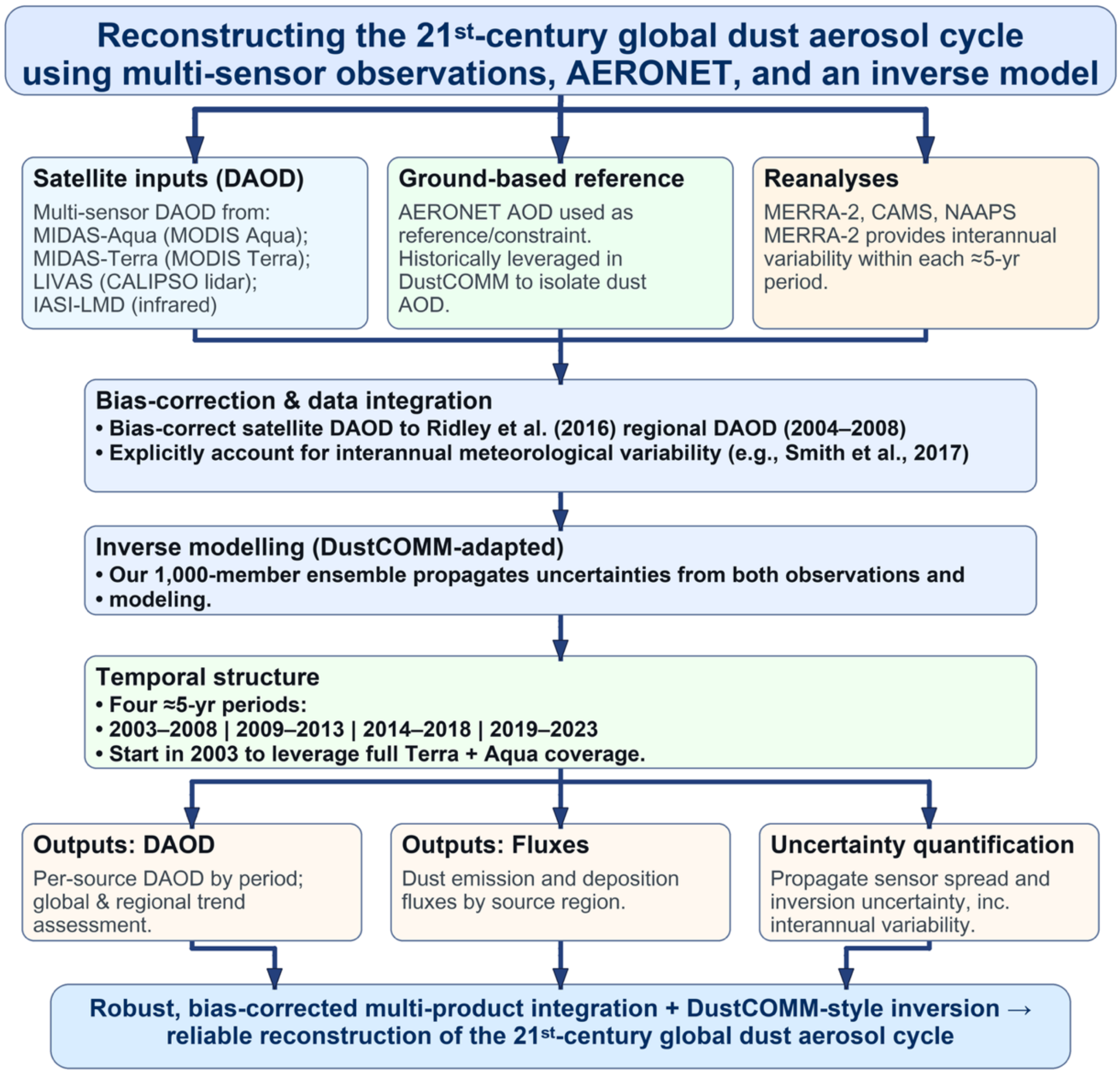

Figure S3: Methodological framework for reconstructing the 21st century global dust aerosol cycle using satellite, reanalysis, and ground-based observations integrated with an inverse model. *The framework integrates multiple satellite-based aerosol datasets (LIVAS, MIDAS-Aqua, MIDAS-Terra, IASI-LMD), ground-based AERONET observations (e.g., Ridley et al., 2016), and three global aerosol reanalysis products (NAAPS, CAMS, MERRA-2). Satellite-derived DAOD trends were evaluated against AERONET coarse-mode AOD observations. Seasonal regional DAOD was extracted from these data sets for 11 major dust-dominated regions and 4 less dusty regions (**fig. S1**) and used in the DustCOMM inverse model to obtain DAOD, dust loading, dust emission fluxes, and deposition fluxes from 2003 to 2023. Uncertainties were assessed through the spread of the ensemble of 1000 different bootstrap simulations (see Materials and Methods).*

Figure S4

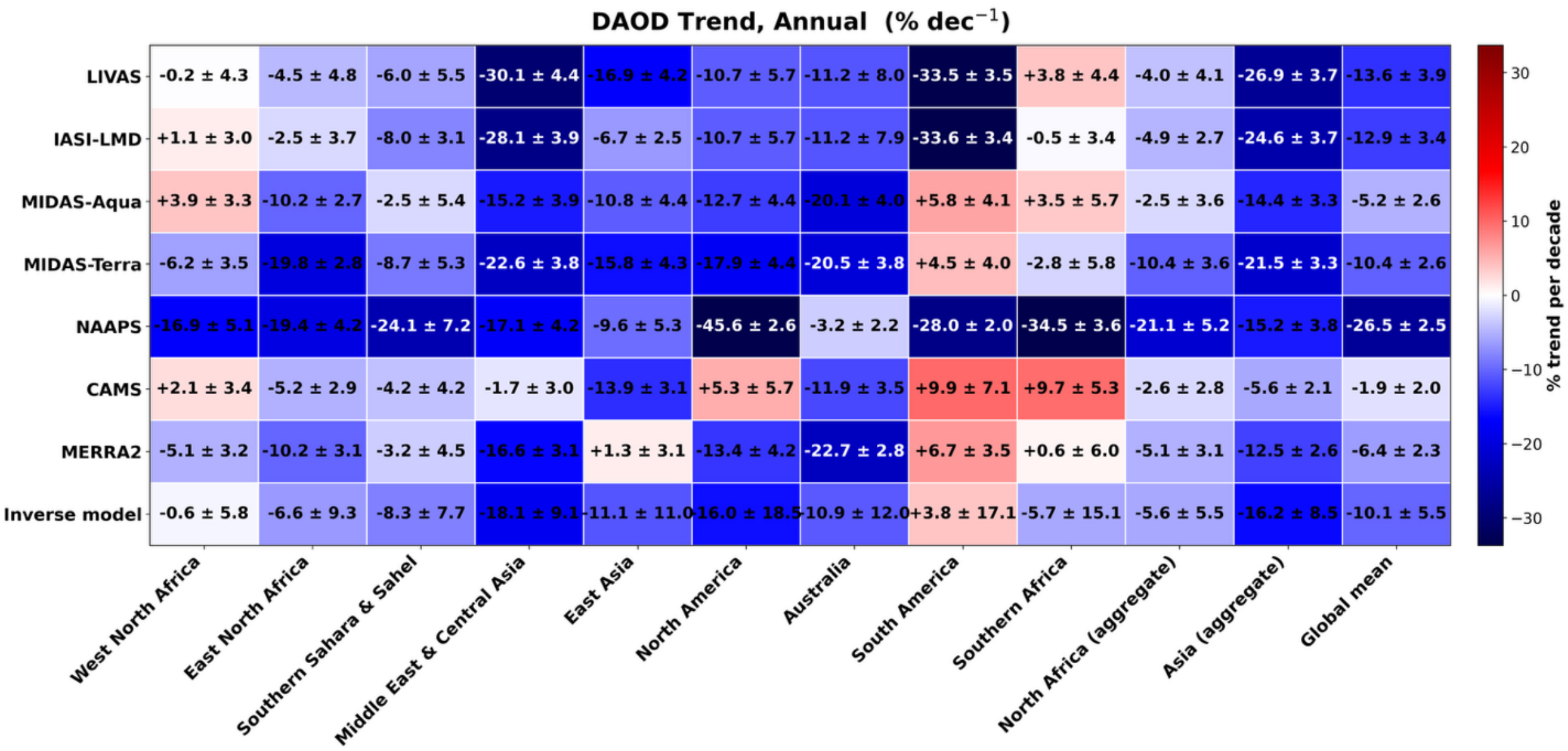

Figure S4: Trends in annual-mean dust aerosol optical depth (DAOD) over 2008–2021. *Heat map of linear trends in annual-mean DAOD for 2008–2021, shown as percentage change per decade (%*dec^{-1}*) relative to the 2008–2021 mean. Columns show regions (global mean at right) and rows show DAOD products (LIVAS, IASI–LMD, MIDAS, MIDAS–Terra, NAAPS, CAMS, MERRA-2 and the inverse-model estimate). Numbers in each cell indicate the regional trend with its one standard error.*

Figure S5

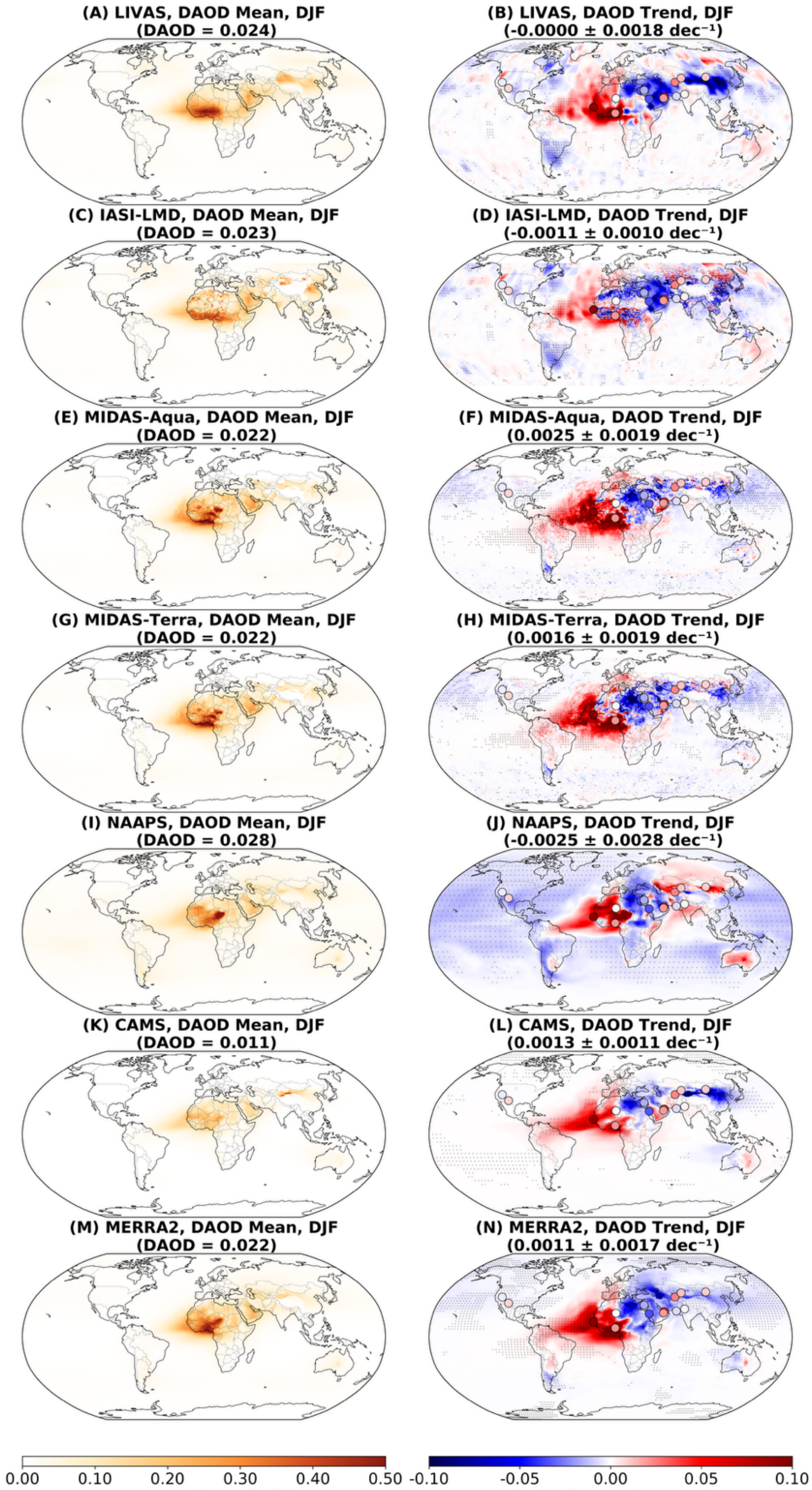

Figure S5: Global dust aerosol optical depth (DAOD) climatology and trends from satellite, reanalysis, and AERONET data during Northern Winter (DJF) season. *Panels (**A, C, E, G, I, K, and M**) show the annual mean DAOD at 550 nm for the overlapping period 2008-2021 from*

*LIVAS (CALIPSO), IASI-LMD, MIDAS-Aqua, MIDAS-Terra, NAAPS, CAMS-RA, and MERRA-2. Panels (**B, D, F, H, J, L, and N)** display the corresponding DAOD linear trends (decade^{-1}). Circles in (**B, D, F, H, J, L, and N)** show the linear trends of conditionally filtered (see Methods) coarse-mode aerosol optical depth (AOD) derived from AERONET sun-photometer records at individual stations, as listed in table S1. Global area-weighted means and decadal trends are reported above each map. Small grey dots indicate grid cells where the linear DAOD trend is statistically significant at the 95% confidence level ($p < 0.05$).*

Figure S6

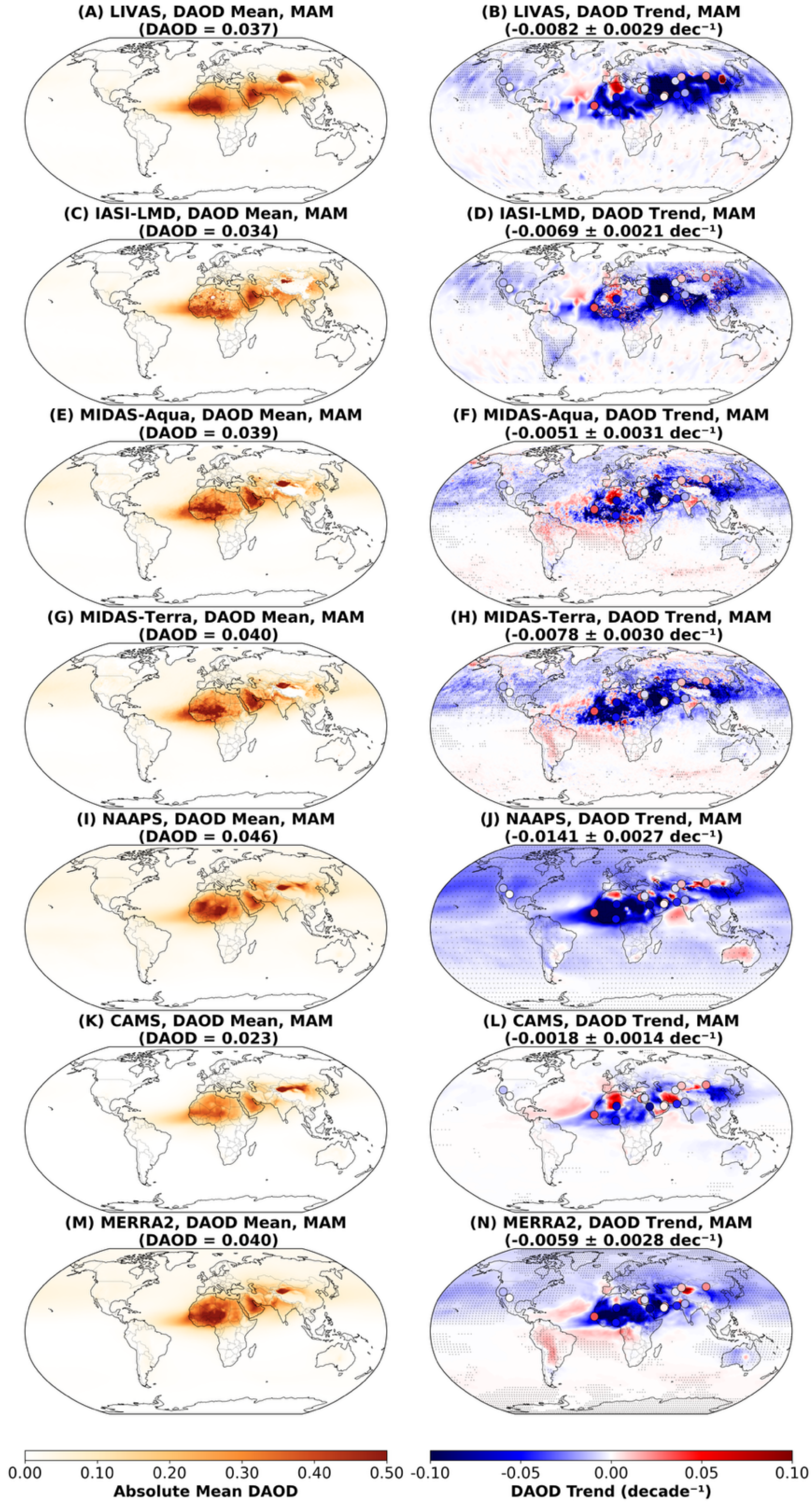

Figure S6: Global dust aerosol optical depth (DAOD) climatology and trends from satellite, reanalysis, and AERONET data during Northern Spring (MAM) season. *Panels (**A, C, E, G, I, K, and M**) show the annual mean DAOD at 550 nm for the overlapping period 2008-2021 from*

LIVAS (CALIPSO), IASI-LMD, MIDAS-Aqua, MIDAS-Terra, NAAPS, CAMS-RA, and MERRA-2. Panels ***(B, D, F, H, J, L, and N)*** *display the corresponding DAOD linear trends (decade^{-1}). Circles in* ***(B, D, F, H, J, L, and N)*** *show the linear trends of conditionally filtered (see Methods) coarse-mode aerosol optical depth (AOD) derived from AERONET sun-photometer records at individual stations, as listed in table S1. Global area-weighted means and decadal trends are reported above each map. Small grey dots indicate grid cells where the linear DAOD trend is statistically significant at the 95% confidence level ($p < 0.05$).*

Figure S7

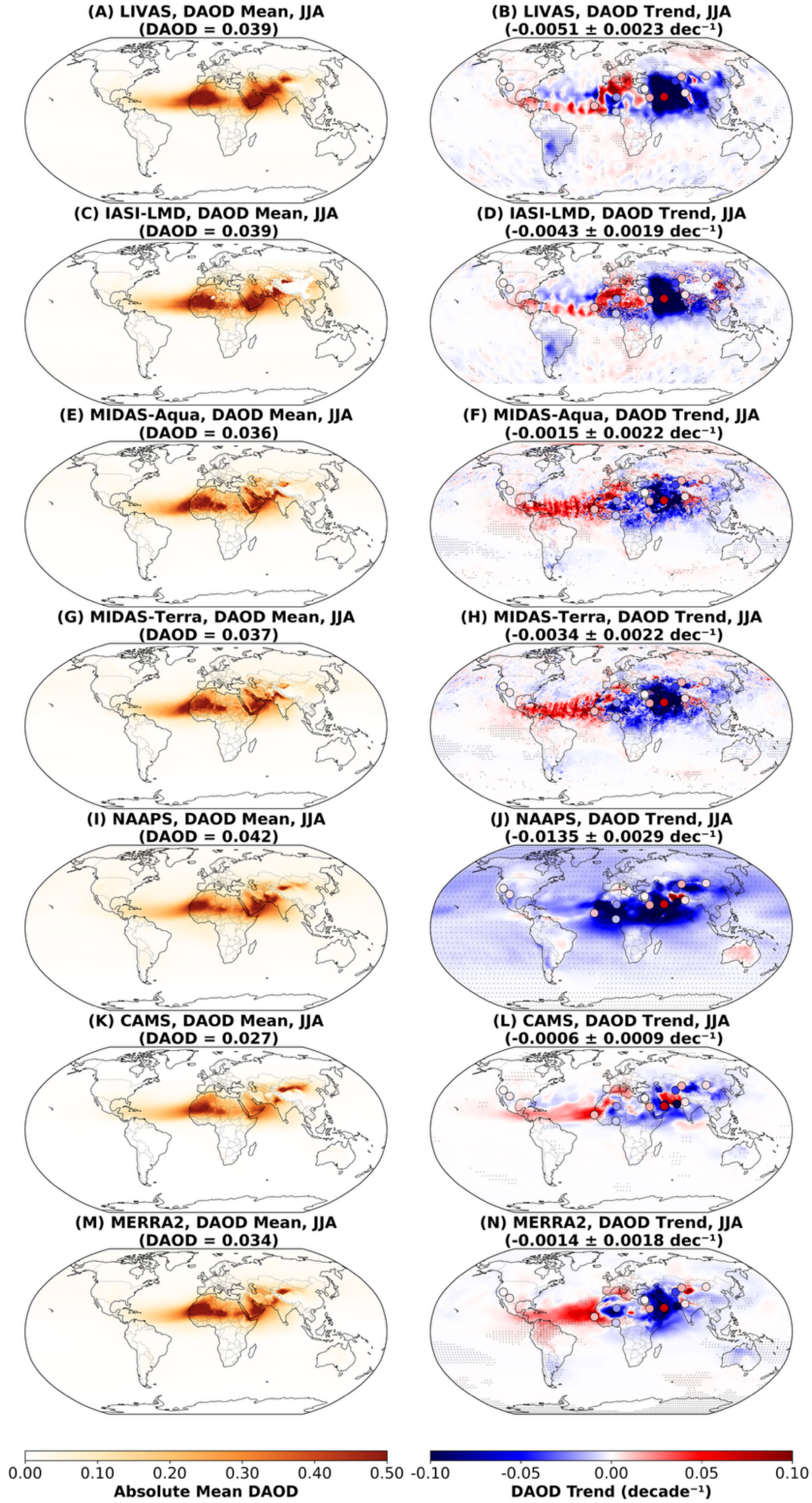

Figure S7: Global dust aerosol optical depth (DAOD) climatology and trends from satellite, reanalysis, and AERONET data during Northern Summer (JJA) season. *Panels (**A, C, E, G, I,***

K, and M*) show the annual mean DAOD at 550 nm for the overlapping period 2008-2021 from LIVAS (CALIPSO), IASI-LMD, MIDAS-Aqua, MIDAS-Terra, NAAPS, CAMS-RA, and MERRA-2. Panels (***B, D, F, H, J, L, and N)*** display the corresponding DAOD linear trends (decade^{-1}). Circles in (***B, D, F, H, J, L, and N)*** show the linear trends of conditionally filtered (see Methods) coarse-mode aerosol optical depth (AOD) derived from AERONET sun-photometer records at individual stations, as listed in table S1. Small grey dots indicate grid cells where the linear DAOD trend is statistically significant at the 95% confidence level ($p < 0.05$).*

Figure S8

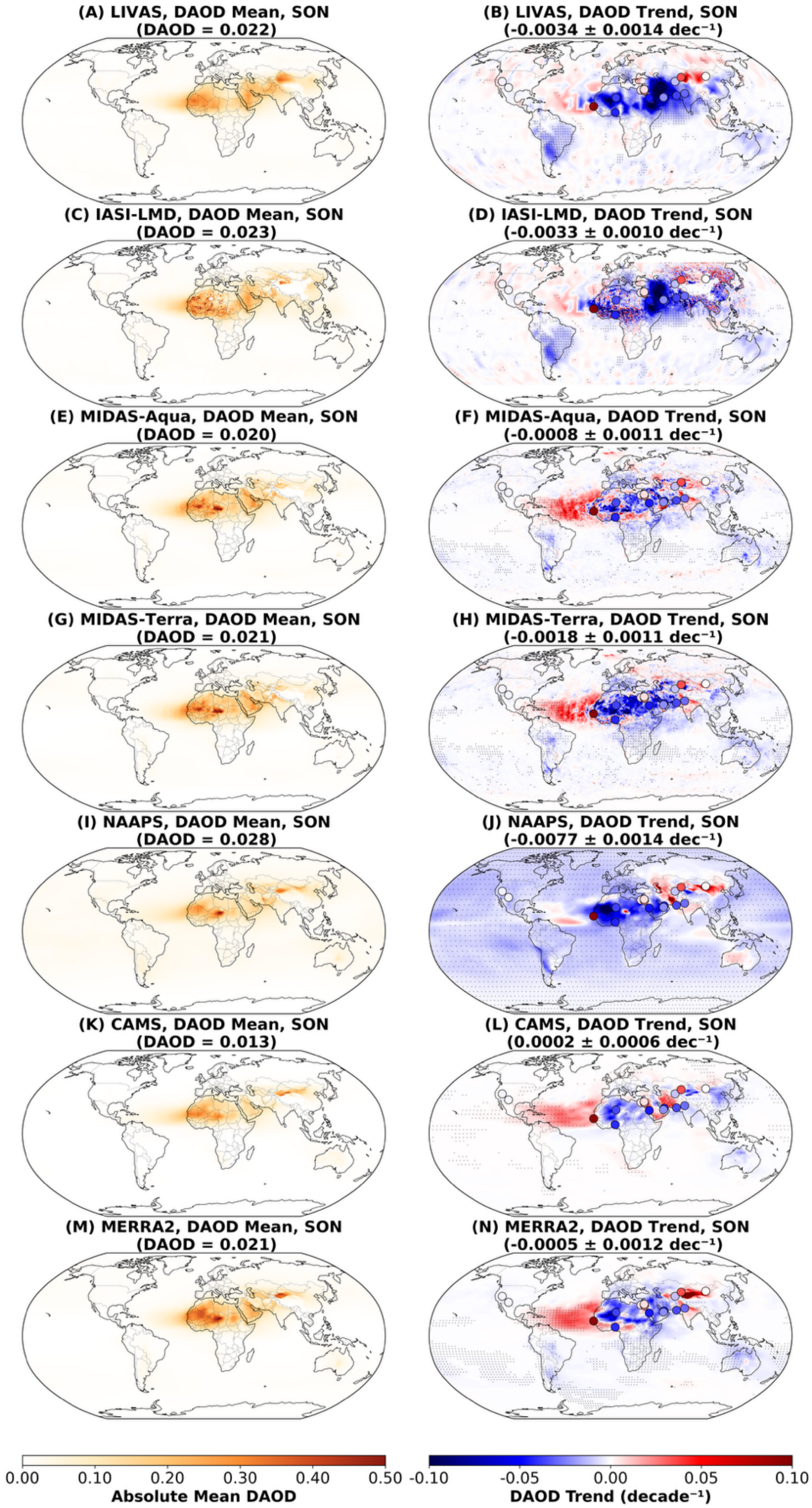

Figure S8: Global dust aerosol optical depth (DAOD) climatology and trends from satellite, reanalysis, and AERONET data during Northern Autumn (SON) season. *Panels (**A, C, E, G,***

*I, K, and M) show the annual-mean DAOD at 550 nm for the overlapping period 2008-2021 from LIVAS (CALIPSO), IASI-LMD, MIDAS-Aqua, MIDAS-Terra, NAAPS, CAMS-RA, and MERRA-2. Panels **(B, D, F, H, J, L, and N)** display the corresponding DAOD linear trends (decade^{-1}). Circles in **(B, D, F, H, J, L, and N)** show the linear trends of conditionally filtered (see Methods) coarse-mode aerosol optical depth (AOD) derived from AERONET sun-photometer records at individual stations, as listed in Table S1. Small grey dots indicate grid cells where the linear DAOD trend is statistically significant at the 95% confidence level ($p < 0.05$).*

Figure S9

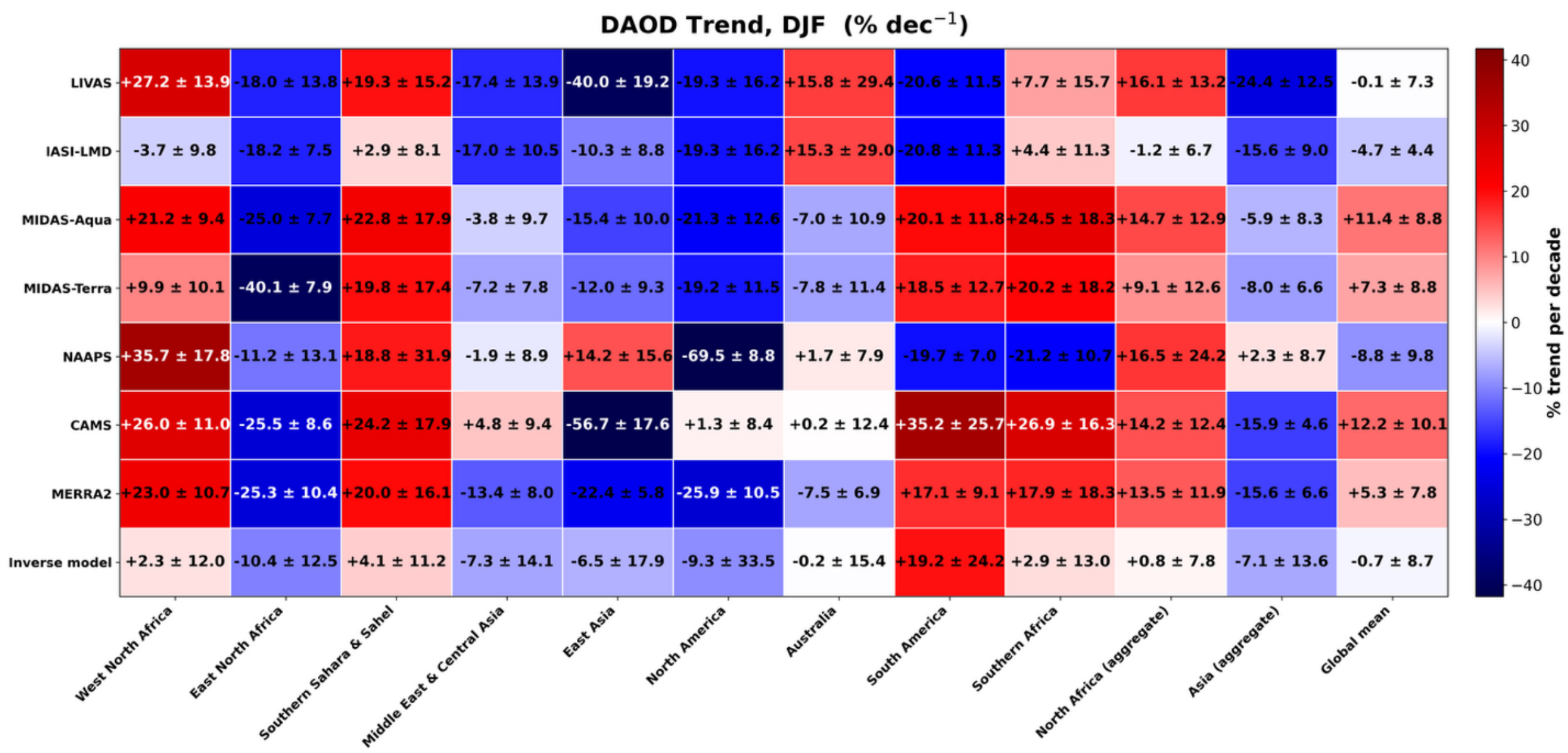

Figure S9: Trends in Northern winter dust aerosol optical depth (DAOD) over 2008–2021. *Heat map of linear trends in Northern winter DAOD for 2008–2021, shown as percentage change per decade (%dec^{-1}) relative to the 2008–2021 mean.*

Figure S10

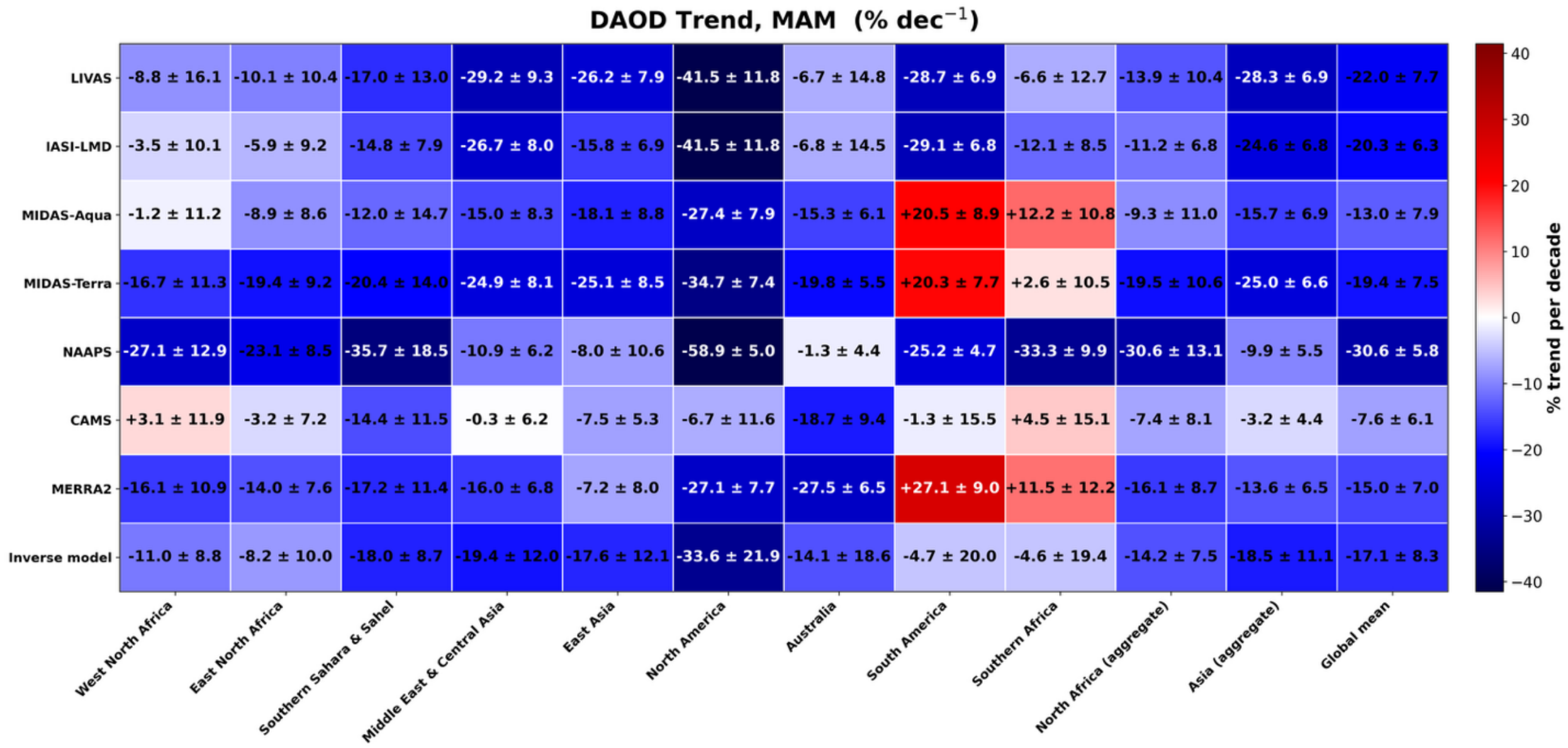

Figure S10: Trends in Northern spring (MAM) dust aerosol optical depth (DAOD) over 2008–2021. *Heat map of linear trends in Northern* ***spring*** *DAOD for 2008–2021, shown as percentage change per decade (%*dec^{-1}*) relative to the 2008–2021 mean.*

Supplementary *Figure 11*

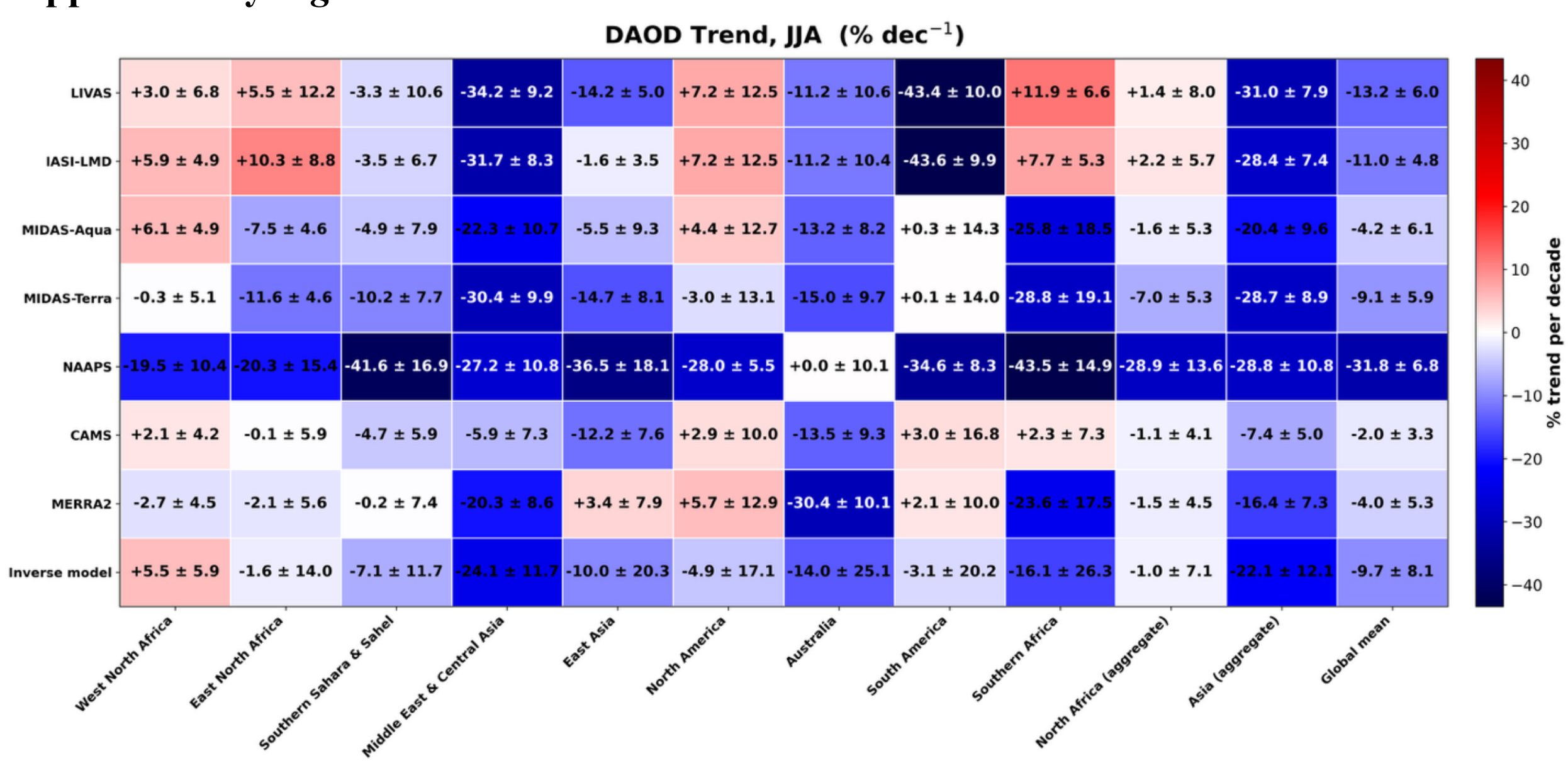

Figure S11: Trends in Northern summer (JJA) dust aerosol optical depth (DAOD) over 2008–2021. *Heat map of linear trends in Northern* ***summer*** *DAOD for 2008–2021, shown as percentage change per decade (%*dec^{-1}*) relative to the 2008–2021 mean.*

Figure S12

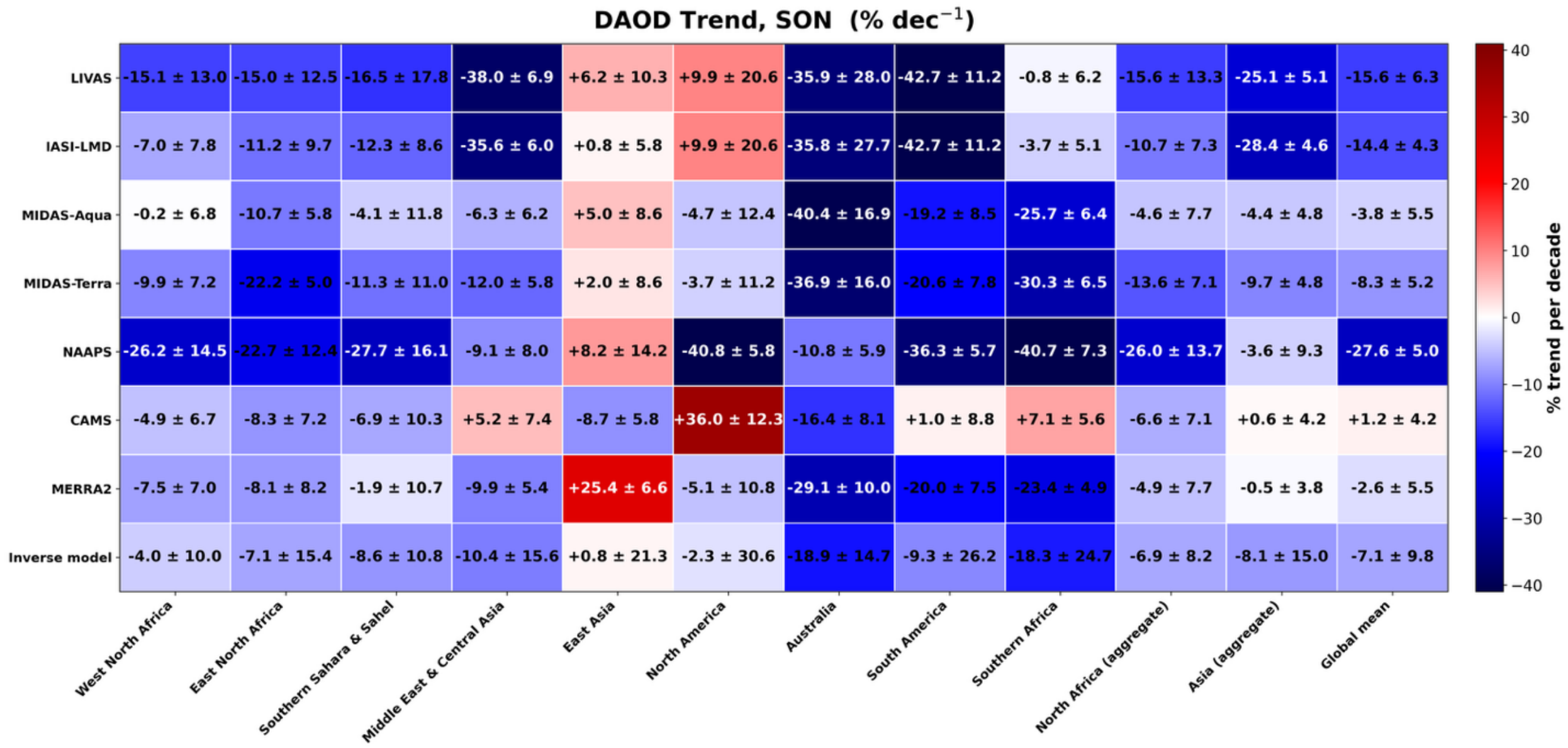

Figure S12: Trends in Northern autumn (SON) dust aerosol optical depth (DAOD) over 2008–2021. *Heat map of linear trends in Northern* ***autumn*** *DAOD for 2008–2021, shown as percentage change per decade (%dec^{-1}) relative to the 2008–2021 mean.*

Figure S13

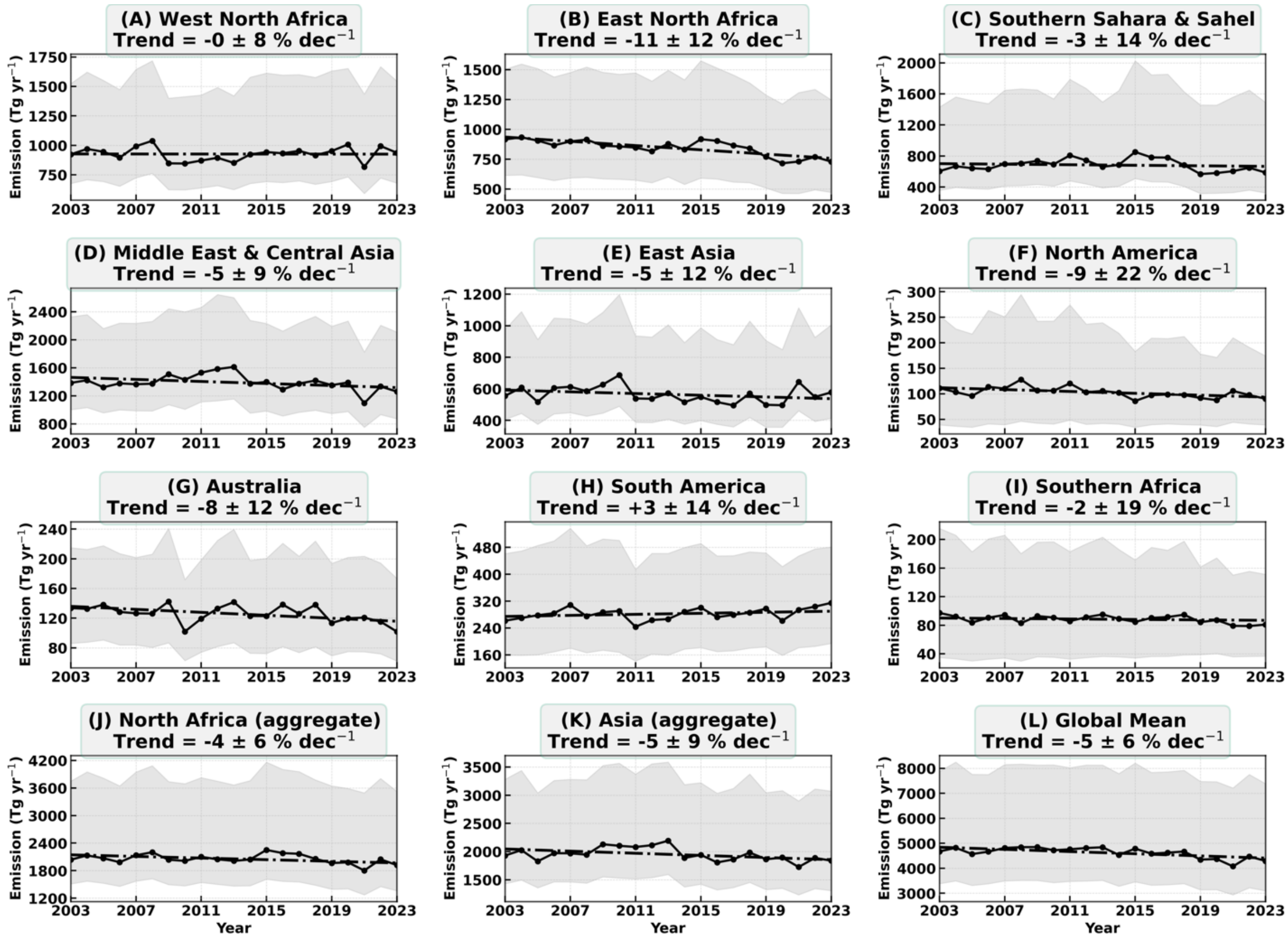

Figure S13. Inverse-model–based reconstruction of regional and global dust emissions, 2003–2023*: Annual inverse-model–based dust emission fluxes from 2003 to 2023 in nine source regions (**panels A–I**), two aggregated domains over North Africa and Asia (**panels J–K**), and all nine sources combined (**panel L**). The black line represents the inverse-model median of dust emission (Tg yr^{-1}). The brown line represents the relative changes in the inverse-model median of dust emission (Tg yr^{-1}), with shading indicating the ±1σ (16th–84th percentile;* P16–P84*) range. The gray dashed-dotted line indicates the trend, and the inset panel shows the estimated trend per decade based on the relative changes in the inverse-model median of dust emission from 2003 to 2023.*

Figure S14

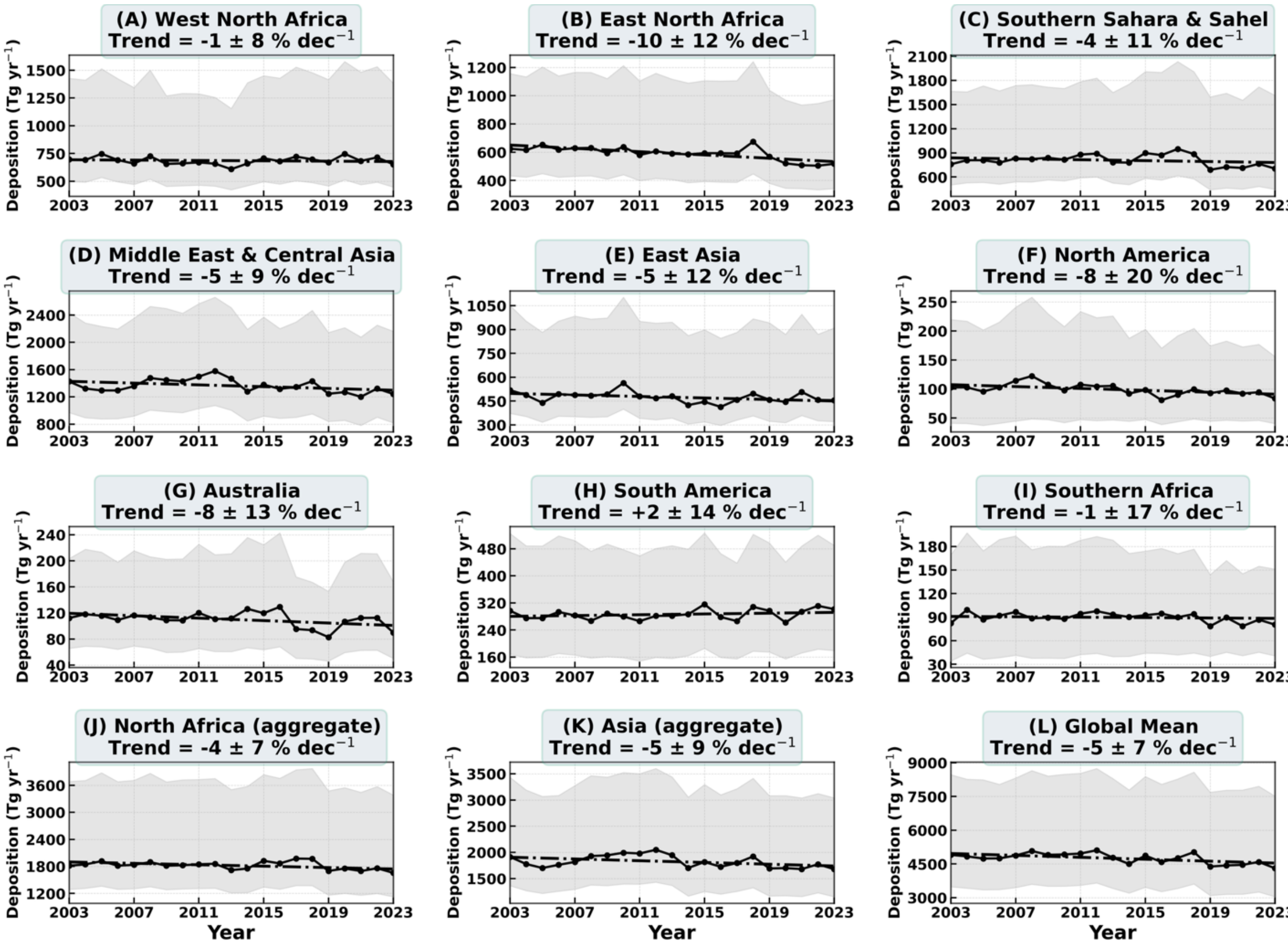

Figure S14. Inverse-model–based reconstruction of regional and global dust deposition fluxes, 2003–2023*: Annual inverse-model–based dust deposition from 2003 to 2023 in nine source regions (**panels A–I**), two aggregated domains over North Africa and Asia (**panels J–K**), and over globe (**panel L**). The black line represents the inverse-model median of dust deposition (Tg yr^{-1}). The brown line represents the relative changes in the inverse-model median of dust deposition (Tg yr^{-1}), with shading indicating the ±1σ (16th–84th percentile; P16–P84) range. The gray dashed-dotted line indicates the trend, and the inset panel shows the estimated trend per decade based on the relative changes in the inverse-model median of dust deposition from 2003 to 2023. Note that the deposition values here represent the flux of dust depositing within each region, not dust originating from each region.*

Figure S15

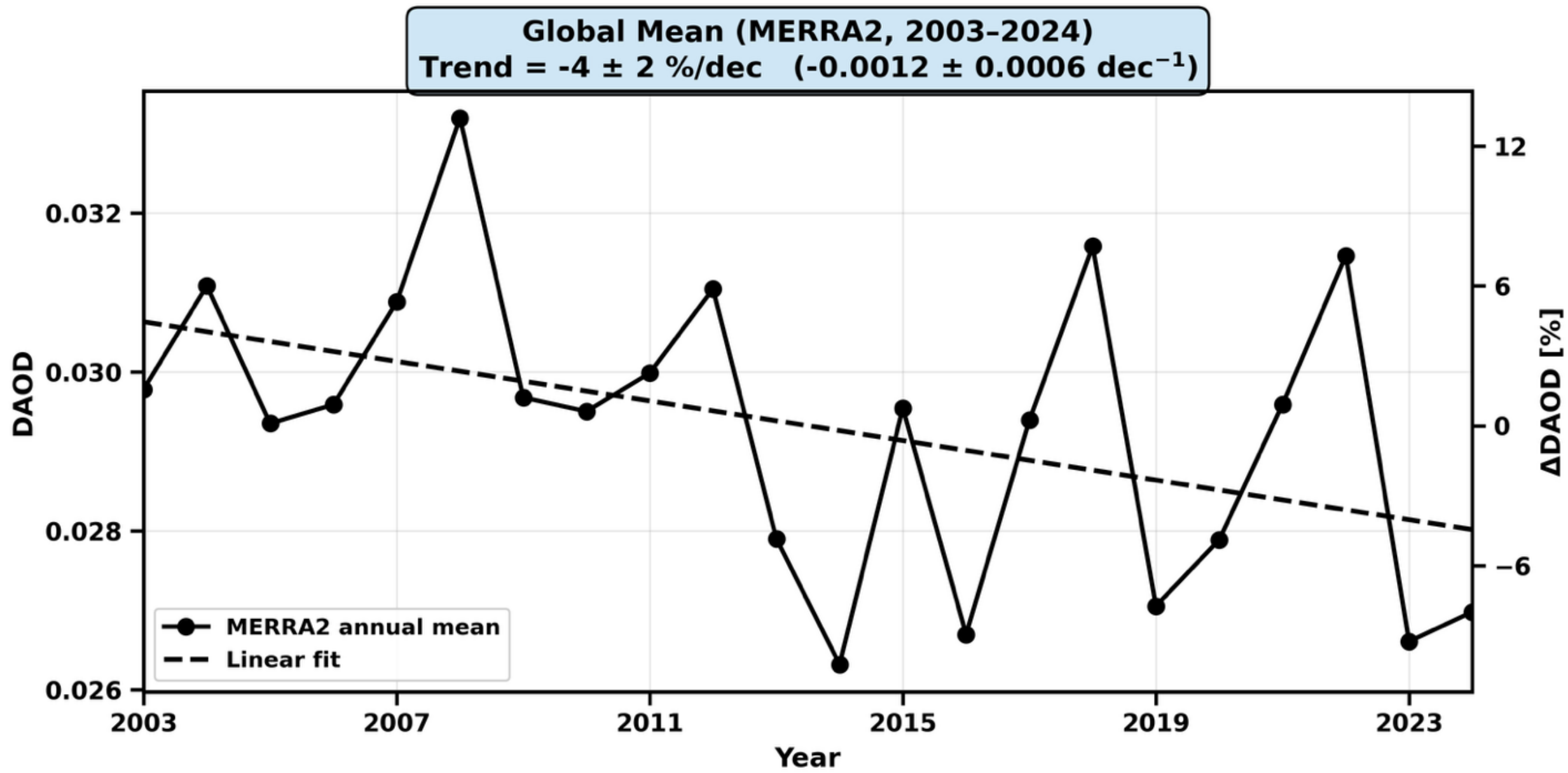

Figure S15: Global mean MERRA-2 DAOD, 2003–2024. *Annual global area-weighted mean DAOD (black circles) and linear trend (dashed line). The trend is* -0.0012 ± 0.0006 *dec*$^{-1}$ *(*-4 ± 2 *% per decade), confirming the negative trend reported over 2003–2024 (Fig. 1).*

Figure S16

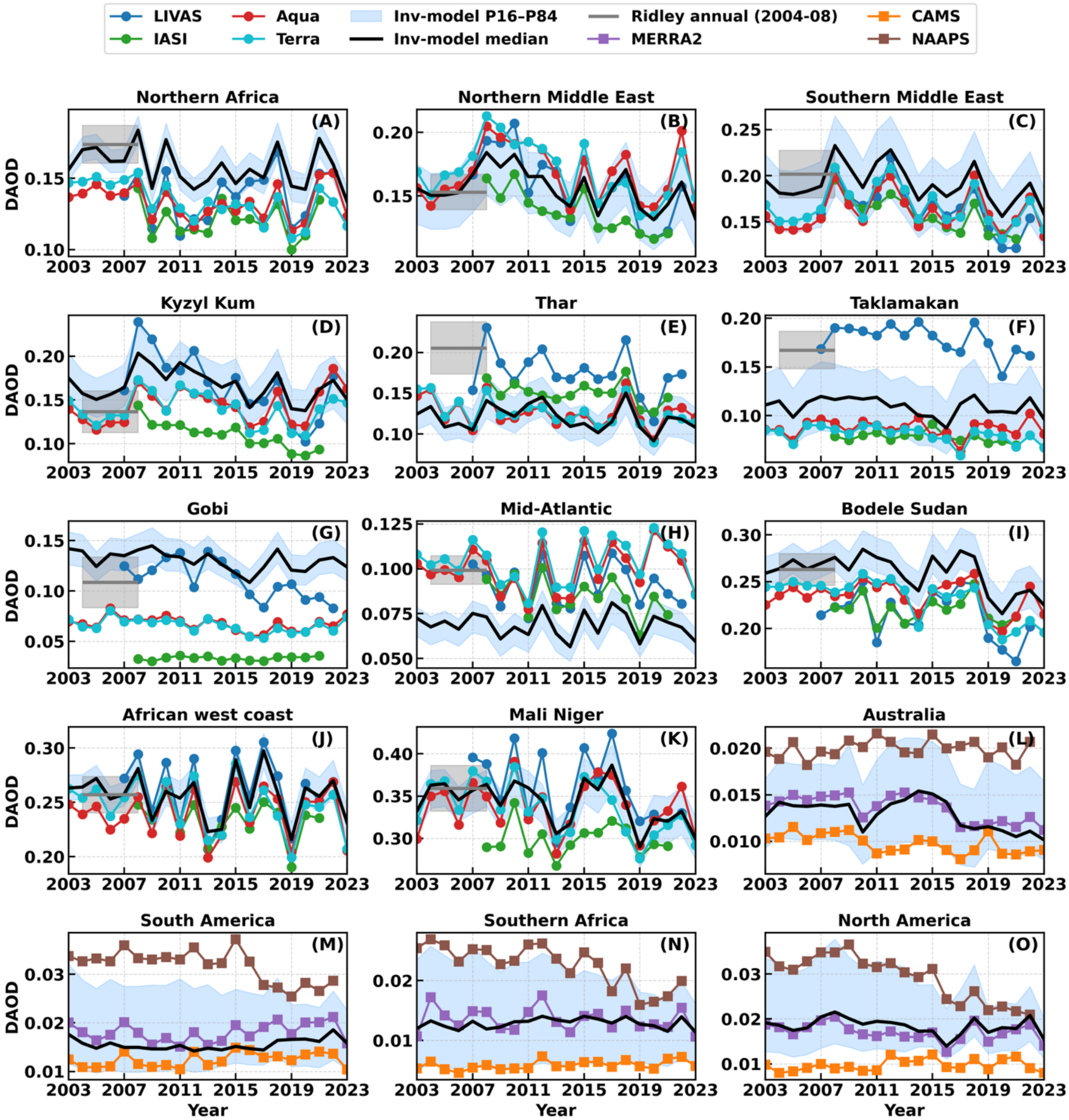

Figure S16. Regional annual dust aerosol optical depth (DAOD) time series and inverse model results (2003–2023) *for the 15 dusty regions. Observations are plotted as solid lines for LIVAS (blue circles), IASI (green circles), Aqua (red circles), and Terra (cyan circles). Reanalysis products are plotted as solid lines for MERRA-2 (purple squares), CAMS (orange squares), and NAAPS (brown squares). The inverse-model reconstruction is represented by the ensemble median (black line), with blue shading bands denoting the ±1σ (16th–84th percentile) range. For selected regions, seasonal and annual means from Ridley et al. (2016) for 2004–2008 are*

*overlaid as horizontal gray bars with uncertainty envelopes. Panels (**A–O**) correspond to the 15 source regions defined in the Materials and Methods and Figure S1.*

Figure S17

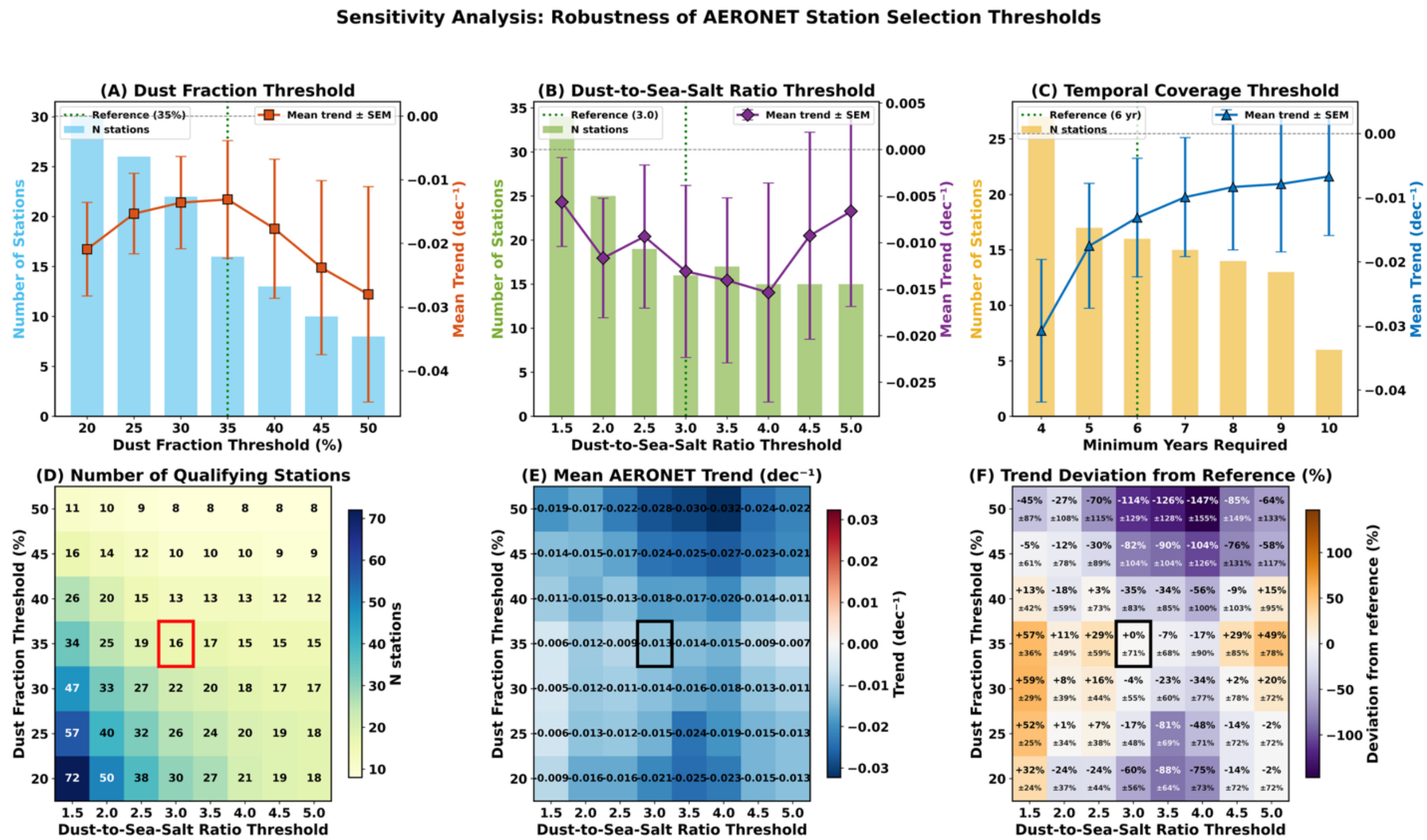

Figure S17: *Sensitivity analysis of the AERONET station selection thresholds. **(A–C)**, One-dimensional cross-section in which each of the three filtering thresholds is varied independently while the other two are held at their reference values (green dotted line): **(A)**, dust fraction threshold (20–50%); **(B)**, MERRA-2 dust-to-sea-salt ratio threshold (1.5–5.0); **(C)**, minimum temporal coverage requirement (4–10 years). Colored bars (left axis) show the number of qualifying AERONET stations at each threshold setting; markers with error bars (right axis) show the mean AERONET coarse-mode AOD trend across qualifying stations, with error bars representing the standard error of the mean (SEM = σ/√N). The horizontal grey dashed line marks zero trend. **(D–F),** Two-dimensional cross-section over the dust fraction × dust-to-sea-salt ratio parameter space (minimum 6 years held fixed at the reference value): **(D)**, number of qualifying stations in each cell; **(e)**, mean AERONET trend (dec⁻¹); **F**, percentage deviation of the mean trend from the reference configuration (top of each cell). The reference cell (dust fraction = 35%, dust-to-sea-salt ratio = 3.0) is outlined in red in **D** and in black in **E** and **F**. The sign of the mean trend is preserved across all 56 tested threshold combinations, and the largest deviations from the reference trend occur at the strictest threshold combinations where the qualifying station count drops sharply; in all such cases the deviation is comparable to or smaller than the SEM, confirming that the apparent variation is consistent with sampling noise from a smaller station ensemble rather than a genuine dependence of the trend on threshold choice.*

Figure S18

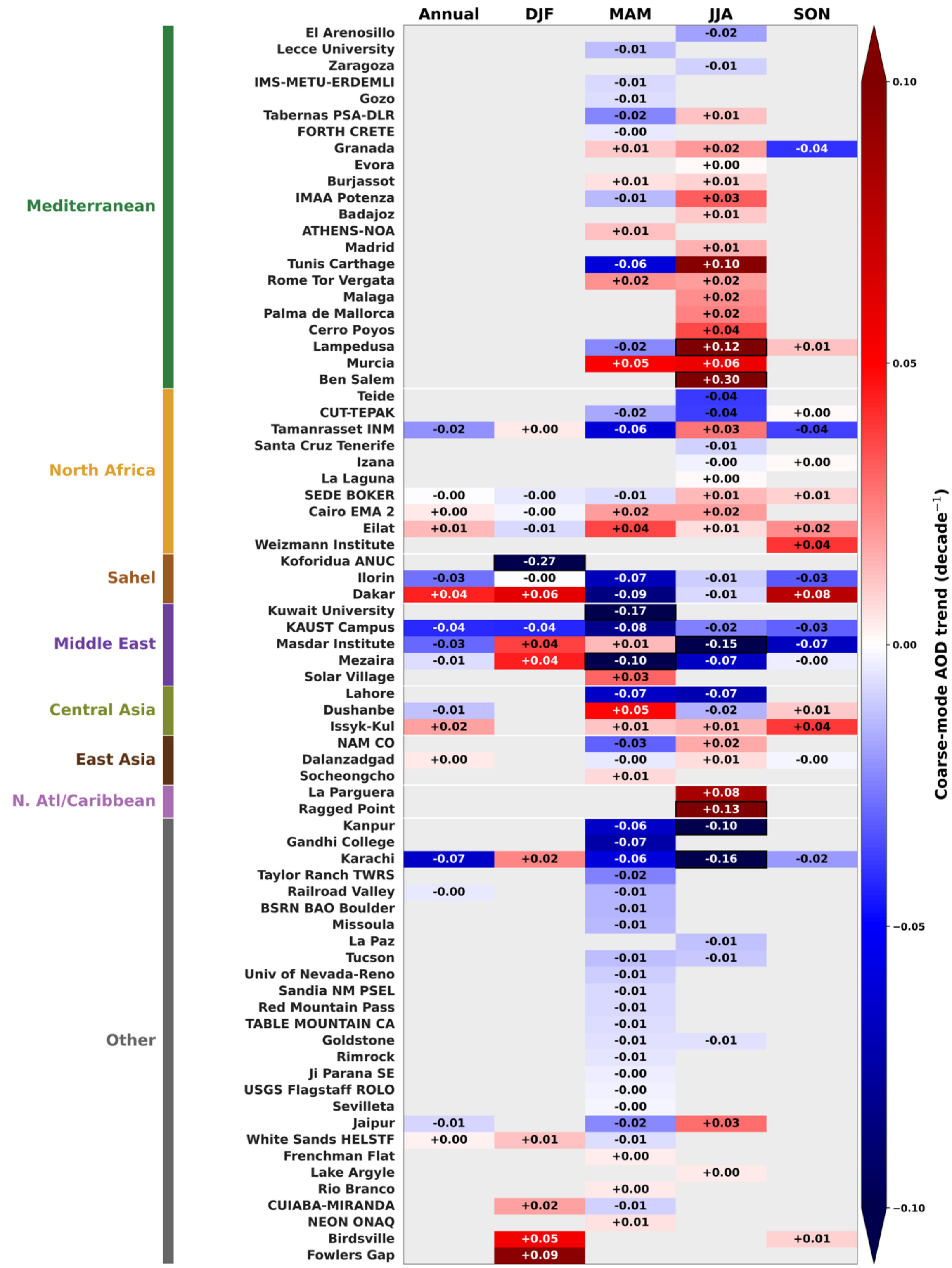

Station	Annual	DJF	MAM	JJA	SON
El Arenosillo				-0.02	
Lecce University			-0.01		
Zaragoza				-0.01	
IMS-METU-ERDEMLI			-0.01		
Gozo			-0.01		
Tabernas PSA-DLR			-0.02	+0.01	
FORTH CRETE			-0.00		
Granada			+0.01	+0.02	-0.04
Evora				+0.00	
Burjassot			+0.01	+0.01	
IMAA Potenza			-0.01	+0.03	
Badajoz				+0.01	
ATHENS-NOA			+0.01		
Madrid				+0.01	
Tunis Carthage			-0.06	+0.10	
Rome Tor Vergata			+0.02	+0.02	
Malaga				+0.02	
Palma de Mallorca				+0.02	
Cerro Poyos				+0.04	
Lampedusa			-0.02	+0.12	+0.01
Murcia			+0.05	+0.06	
Ben Salem				+0.30	
Teide				-0.04	
CUT-TEPAK			-0.02	-0.04	+0.00
Tamanrasset INM	-0.02	+0.00	-0.06	+0.03	-0.04
Santa Cruz Tenerife				-0.01	
Izana				-0.00	+0.00
La Laguna				+0.00	
SEDE BOKER	-0.00	-0.00	-0.01	+0.01	+0.01
Cairo EMA 2	+0.00	-0.00	+0.02	+0.02	
Eilat	+0.01	-0.01	+0.04	+0.01	+0.02
Weizmann Institute					+0.04
Koforidua ANUC		-0.27			
Ilorin	-0.03	-0.00	-0.07	-0.01	-0.03
Dakar	+0.04	+0.06	-0.09	-0.01	+0.08
Kuwait University			-0.17		
KAUST Campus	-0.04	-0.04	-0.08	-0.02	-0.03
Masdar Institute	-0.03	+0.04	+0.01	-0.15	-0.07
Mezaira	-0.01	+0.04	-0.10	-0.07	-0.00
Solar Village			+0.03		
Lahore			-0.07	-0.07	
Dushanbe	-0.01		+0.05	-0.02	+0.01
Issyk-Kul	+0.02		+0.01	+0.01	+0.04
NAM CO			-0.03	+0.02	
Dalanzadgad	+0.00		-0.00	+0.01	-0.00
Socheongcho			+0.01		
La Parguera				+0.08	
Ragged Point				+0.13	
Kanpur			-0.06	-0.10	
Gandhi College			-0.07		
Karachi	-0.07	+0.02	-0.06	-0.16	-0.02
Taylor Ranch TWRS			-0.02		
Railroad Valley	-0.00		-0.01		
BSRN BAO Boulder			-0.01		
Missoula			-0.01		
La Paz				-0.01	
Tucson			-0.01	-0.01	
Univ of Nevada-Reno			-0.01		
Sandia NM PSEL			-0.01		
Red Mountain Pass			-0.01		
TABLE MOUNTAIN CA			-0.01		
Goldstone			-0.01	-0.01	
Rimrock			-0.01		
Ji Parana SE			-0.00		
USGS Flagstaff ROLO			-0.00		
Sevilleta			-0.00		
Jaipur	-0.01		-0.02	+0.03	
White Sands HELSTF	+0.00	+0.01	-0.01		
Frenchman Flat			+0.00		
Lake Argyle				+0.00	
Rio Branco			+0.00		
CUIABA-MIRANDA		+0.02	-0.01		
NEON ONAQ			+0.01		
Birdsville		+0.05			+0.01
Fowlers Gap		+0.09			

***Figure S18**: **Seasonal trends in coarse-mode AOD at 3C-filtered AERONET stations.** Rows, individual stations grouped by region; columns, annual mean and four seasons. Cell color shows*

the linear trend (decade⁻¹) in coarse-mode AOD; grey cells indicate seasons in which the station does not pass the 3C qualification criteria. Black outlines mark trends that exceed the color-bar range (±0.10 decade⁻¹). Of 75 stations qualifying in at least one season, 16 also pass the annual filter.

Figure S19

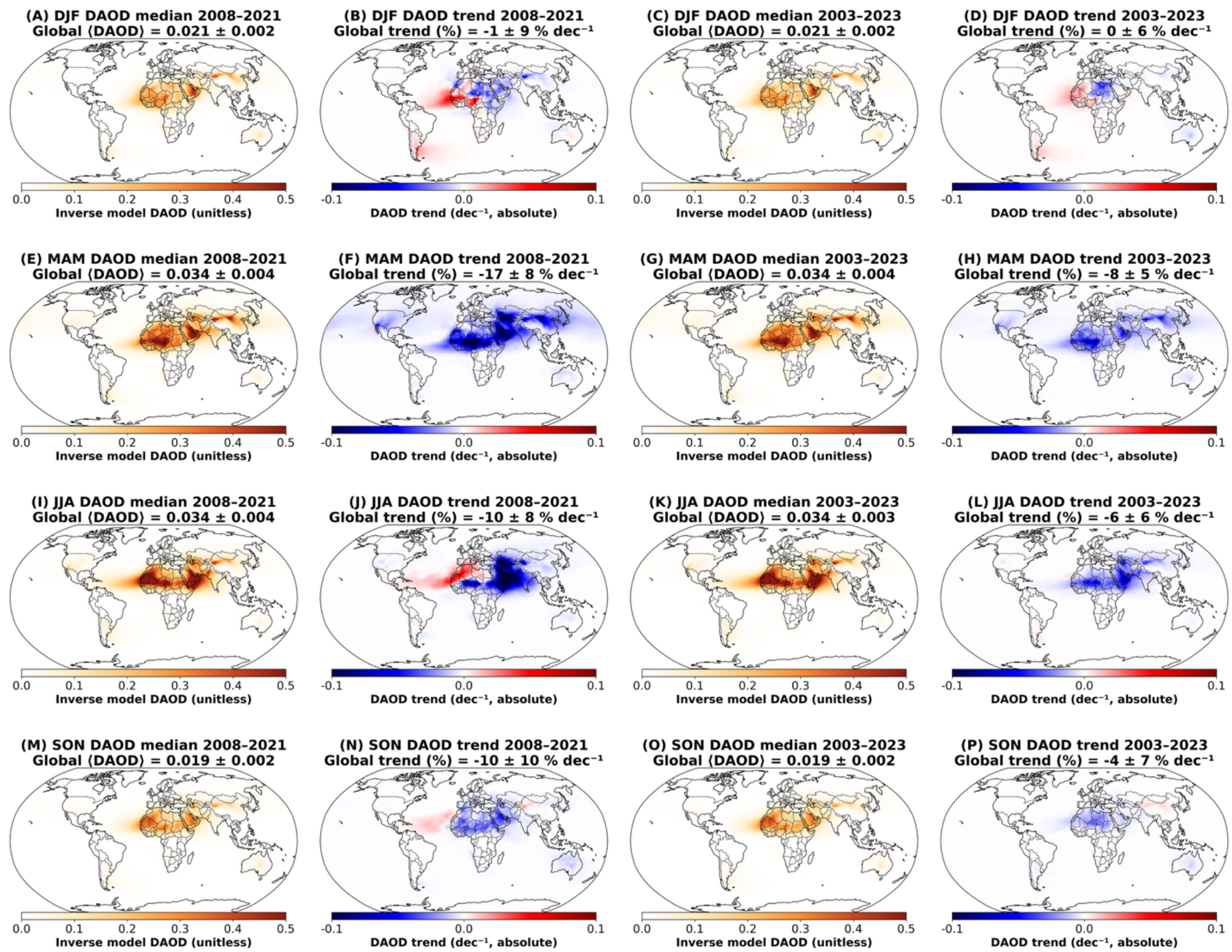

***Figure S19: Seasonal global DAOD from the inverse-model ensemble.** Ensemble-median time-mean DAOD (columns 1, 3) and per-pixel linear trend (columns 2, 4) for boreal winter (DJF; **A–D**), spring (MAM; **E–H**), summer (JJA; **I–L**) and autumn (SON; **M–P**), evaluated over 2008–2021 (columns 1–2) and 2003–2023 (columns 3–4). Trends are ordinary least-squares slopes on the member-median seasonal series, in absolute units of dec⁻¹. Panel titles report the area-weighted global mean DAOD (mean panels) and the global relative trend in % dec⁻¹ (trend panels), each given as the ensemble median ± half the (P16, P84) inter-percentile range across members.*

table S1

Datasets	LIVAS	MIDAS-Aqua-PM/Terra-AM	IASI-LMD	NAAPS	CAMS	MERRA-2
Time Period	**2007-2022**	**2003-2023**	**2008-2021**	**2003-2023**	**2003-2023**	**2003-2023**
**Spatial/ Temporal Resolution**	**Horizontal: 1°** (Vertical: ~60 m bins below 20 km)	**~0.1°** (~10 km), daily swaths	**Footprints ~12 km** Daily mapping ~0.5° (with interpolation)	**~1° grid** 6-hourly outputs	**~0.7° grid** 3-hourly outputs	**~0.5° grid** 3-hourly outputs
**Coverage**	**Global but sparse** (narrow orbital track); monthly averaging often needed)	**Global, wide-swath** coverage (1–2 overpasses per day	**Global** (2 overpasses per day), nighttime retrievals possible	**Global, continuous** coverage (no data gaps-model filled)	**Global** coverage with continuous fields over multiple years	Global coverage extends back to 1980 (long record)

table S1. Comparison of aerosol datasets and their characteristics for six DAOD products (LIVAS, MIDAS-Aqua-PM/Terra-AM, IASI-LMD, NAAPS, CAMS, MERRA-2). *We detail the available time periods, spatial and temporal resolutions (ranging from coarse ~0.5°-1° grids and narrow orbital tracks to high-resolution daily swaths and 3-hourly outputs), global coverage nuances, and associated accuracy and uncertainty metrics, including comparisons to AERONET measurements. Note that we used a harmonized 0.5° × 0.5° grid for all seven products as input to the inverse model.*

table S2

	Station	Latitude (°)	Longitude (°)	Slope ± stderr (decade⁻¹)	Region
1	Dushanbe	38.5533	68.8579	-0.0120 ± 0.0139	Central Asia
2	Issyk-Kul	42.6228	76.9831	+0.0184 ± 0.0070	Central Asia
3	Dalanzadgad	43.5772	104.4192	+0.0045 ± 0.0047	East Asia
4	KAUST Campus	22.3048	39.1028	-0.0414 ± 0.0249	Middle East
5	Masdar Institute	24.4416	54.6166	-0.0296 ± 0.0249	Middle East
6	Mezaira	23.1045	53.7547	-0.0065 ± 0.0210	Middle East
7	Cairo EMA 2	30.0808	31.2901	+0.0044 ± 0.0110	North Africa
8	Eilat	29.5025	34.9175	+0.0138 ± 0.0172	North Africa
9	SEDE BOKER	30.8550	34.7822	-0.0006 ± 0.0067	North Africa
10	Tamanrasset INM	22.7900	5.5300	-0.0216 ± 0.0162	North Africa
11	Dakar	14.3942	-16.9586	+0.0437 ± 0.0267	Sahel
12	Ilorin	8.4841	4.6745	-0.0281 ± 0.0252	Sahel
13	Jaipur	26.9058	75.8062	-0.0081 ± 0.0197	Other
14	Karachi	24.9457	67.1359	-0.0652 ± 0.0215	Other
15	Railroad Valley	38.4970	-115.6910	-0.0043 ± 0.0019	Other
16	White Sands HELSTF	32.6349	-106.3381	+0.0025 ± 0.0033	Other

table S2: Qualifying AERONET stations and 2008–2021 coarse-mode AOD trends. AERONET *Stations passed 3C-filtered tests: mean dust fraction ≥ 35%, dust/sea-salt mass ratio > 3, and ≥ 6 years with ≥ 8 valid months. Slopes and standard errors (decade⁻¹) are from ordinary least-squares regression on deseasonalized monthly anomalies.*
